# Genomic Insights Into the *Mycobacterium kansasii* Complex: An Update

**DOI:** 10.3389/fmicb.2019.02918

**Published:** 2020-01-15

**Authors:** Tomasz Jagielski, Paulina Borówka, Zofia Bakuła, Jakub Lach, Błażej Marciniak, Anna Brzostek, Jarosław Dziadek, Mikołaj Dziurzyński, Lian Pennings, Jakko van Ingen, Manca Žolnir-Dovč, Dominik Strapagiel

**Affiliations:** ^1^Department of Applied Microbiology, Faculty of Biology, Institute of Microbiology, University of Warsaw, Warsaw, Poland; ^2^Biobank Lab, Department of Molecular Biophysics, Faculty of Biology and Environmental Protection, University of Łódź, Łódź, Poland; ^3^Department of Anthropology, Faculty of Biology and Environmental Protection, University of Łódź, Łódź, Poland; ^4^BBMRI.pl Consortium, Wroclaw, Poland; ^5^Institute of Medical Biology, Polish Academy of Sciences, Łódź, Poland; ^6^Department of Bacterial Genetics, Faculty of Biology, Institute of Microbiology, University of Warsaw, Warsaw, Poland; ^7^Department of Medical Microbiology, Radboud University Medical Center, Nijmegen, Netherlands; ^8^Laboratory for Mycobacteria, University Clinic of Respiratory and Allergic Diseases, Golnik, Slovenia

**Keywords:** *Mycobacterium kansasii* complex, *Mycobacterium ostraviense* sp. nov., non-tuberculous mycobacteria (NTM), whole genome sequencing, taxonomy

## Abstract

Only very recently, has it been proposed that the hitherto existing *Mycobacterium kansasii* subtypes (I–VI) should be elevated, each, to a species rank. Consequently, the former *M. kansasii* subtypes have been denominated as *Mycobacterium kansasii* (former type I), *Mycobacterium persicum* (II), *Mycobacterium pseudokansasii* (III), *Mycobacterium innocens* (V), and *Mycobacterium attenuatum* (VI). The present work extends the recently published findings by using a three-pronged computational strategy, based on the alignment fraction-average nucleotide identity, genome-to-genome distance, and core-genome phylogeny, yet essentially independent and much larger sample, and thus delivers a more refined and complete picture of the *M. kansasii* complex. Furthermore, five canonical taxonomic markers were used, i.e., 16S rRNA, *hsp65, rpoB*, and *tuf* genes, as well as the 16S-23S rRNA intergenic spacer region (ITS). The three major methods produced highly concordant results, corroborating the view that each *M. kansasii* subtype does represent a distinct species. This work not only consolidates the position of five of the currently erected species, but also provides a description of the sixth one, i.e., *Mycobacterium ostraviense* sp. nov. to replace the former subtype IV. By showing a close genetic relatedness, a monophyletic origin, and overlapping phenotypes, our findings support the recognition of the *M. kansasii* complex (MKC), accommodating all *M. kansasii*-derived species and *Mycobacterium gastri*. None of the most commonly used taxonomic markers was shown to accurately distinguish all the MKC species. Likewise, no species-specific phenotypic characteristics were found allowing for species differentiation within the complex, except the non-photochromogenicity of *M. gastri*. To distinguish, most reliably, between the MKC species, and between *M. kansasii* and *M. persicum* in particular, whole-genome-based approaches should be applied. In the absence of clear differences in the distribution of the virulence-associated region of difference 1 genes among the *M. kansasii*-derived species, the pathogenic potential of each of these species can only be speculatively assessed based on their prevalence among the clinically relevant population. Large-scale molecular epidemiological studies are needed to provide a better understanding of the clinical significance and pathobiology of the MKC species. The results of the *in vitro* drug susceptibility profiling emphasize the priority of rifampicin administration in the treatment of MKC-induced infections, while undermining the use of ethambutol, due to a high resistance to this drug.

## Introduction

Non-tuberculous mycobacteria (NTM) comprise all species of the *Mycobacterium* genus, except those aetiologically implicated in tuberculosis (TB) and leprosy, that is members of the *M. tuberculosis* complex and *M. leprae* or *M. lepromatosis*, respectively. More than 180 NTM species have been recognized to date (LPSN database, 2019). This figure, however, will soon need to be revised, since several new species, on average, are continuously being added every year (Tortoli, 2014). With the increasing number of mycobacterial species descriptions, the number of reported infections, essentially due to NTM, is also growing on a global level. Not as much an enlarging spectrum of NTM species, but more a heightened clinical awareness, expanding population of vulnerable hosts, and advancement of diagnostic and surveillance services are responsible for this scenario (Sood and Parrish, 2017). Although many of the newly described NTM species are potentially pathogenic, having been isolated from clinically affected individuals, only less than a third has consistently been associated with significant health disorders in humans.

*Mycobacterium kansasii* is one of the most virulent and prevalent NTM pathogen in human medicine. It was first described by Buhler and Pollak in 1953 from a series of respiratory samples of patients with a TB-like pulmonary disease (Buhler and Pollak, 1953). The species was originally named a “yellow bacillus” to emphasize its brilliant yellow pigmentation on exposure to light, but amended thereupon to *M. tuberculosis luciflavum* by Middlebrook (Middlebrook, 1956) and *M. luciflavum* by Manten (Manten, 1957). The current species name (*M. kansasii*) was proposed by Hauduroy in 1955 and refers to where its first isolations were performed (Kansas City, USA) (Hauduroy, 1955). Since the early 1960s, *M. kansasii* infections have been among the very top of all NTM diseases reported worldwide. A remarkable upsurge in the incidence of *M. kansasii* infections was seen at the turn of 1980s and 1990s, with the burgeoning of the HIV/AIDS epidemic, and persisted until the first antiretroviral therapies became widely available (Horsburgh and Selik, 1989; Witzig et al., 1995; Santin and Alcaide, 2003). Currently, *M. kansasii* is one of the six most frequently isolated NTM species across the world. The prevalence of this pathogen is exceptionally high in Slovakia, Poland, and the UK, with the isolation rate of 36, 35, and 11%, respectively, compared to a mean isolation rate of 5% in Europe and 4% globally (Hoefsloot et al., 2013). Chronic, fibro-cavitary lung disease, with upper lobe predominance, and with an overall clinical picture mimicking classical TB, is the most common manifestation attributable to *M. kansasii* (Matveychuk et al., 2012; Moon et al., 2015; Bakuła et al., 2018b). Much rarer are extrapulmonary infections, such as lymphadenitis, skin and soft-tissue infections, and disseminated disease (Liao et al., 2007; Chen et al., 2008; Park et al., 2012; Shaaban et al., 2014). The exact epidemiology of *M. kansasii* disease is difficult to ascertain because case reporting is not mandatory in most countries and differentiation between isolation (colonization) and infection may be diagnostically challenging. Moreover, the incidence rates are influenced by a combination of demographic and clinical factors, including patient geographical origin and HIV status. Pulmonary *M. kansasii* infections tend to cluster in specific geographical areas, such as central Europe or metropolitan centers of London, Brasilia, and Johannesburg (Hoefsloot et al., 2013). Strong epidemiological disparities exist in terms of HIV reactivity. The annual rate of *M. kansasii* infection among HIV-seropositive patients has been reported to be as high as 532 per 100,000 population, whereas in non-HIV infected individuals it has been calculated at 0.06–2.2 per 100,000 population (Marras and Daley, 2004; Ricketts et al., 2014). Not only the incidence rates, but also the sources of *M. kansasii* infections and routes of transmission are poorly defined. Similar to other NTM, *M. kansasii* infections are believed to be acquired from environmental exposures rather than by person-to-person transmission, although a case of interfamilial clustering has been described (Ricketts et al., 2014). Contrary to other NTM, *M. kansasii* has only sporadically been isolated from soil, natural water systems or animals. Instead, the pathogen has often been recovered from municipal tap water, which is considered its major environmental reservoir (Falkinham, 1996; Thomson et al., 2013).

The genetic structure of *M. kansasii* was first investigated in the early 1990s. A pioneering work by Ross et al. showed the existence of a genetic subspecies of *M. kansasii* by sequencing of the 5′ end of the 16S rRNA gene (Ross et al., 1992). Subsequent studies involving the amplification of the 16S-23S rRNA spacer region (Abed et al., 1995), PCR-restriction analysis (PRA) of the highly-conserved *hsp65* gene (Plikaytis et al., 1992; Telenti et al., 1993) and Southern blot hybridization with the major polymorphic tandem repeat (MPTR) (Hermans et al., 1992) or insertion sequence-like element, IS*1652* (Yang et al., 1993) as a probe have confirmed *M. kansasii* as a genetically heterogeneous species. The genetic variability of *M. kansasii* was clearly demonstrated in a study of Picardeau et al. who divided the species into five subspecies or (sub-)types, based on the analysis of restriction fragment length polymorphisms (RFLPs) using the MPTR probe, pulsed-field gel electrophoresis (PFGE), amplified fragment length polymorphism (AFLP) analysis, and PRA of the *hsp65* gene (Picardeau et al., 1997). The validity of the five *M. kansasii* subtypes was further corroborated by sequencing of the 16S-23S rRNA gene or internal transcribed spacer (ITS) region (Alcaide et al., 1997). Somewhat later, two novel types (VI and VII) have been described, according to their *hsp65* restriction profiles and sequencing results of the 16S rRNA gene and the 16S-23S rRNA spacer (Richter et al., 1999; Taillard et al., 2003). Moreover, *M. kansasii* isolates with an intermediate type I (I/II) and atypical type II (IIb) have been reported. Whereas, the former had type I-specific sequence of the *hsp65* gene and type II-specific sequence of the spacer region, the latter displayed type II-specific spacer sequence and a unique *hsp65* gene sequence (Iwamoto and Saito, 2005). The separateness of the *M. kansasii* subspecies was further supported by polymorphisms at other genetic loci, including the RNA polymerase gene (*rpoB*) and the translational elongation factor Tu (*tuf*), successfully applied for the differentiation between the subspecies I-VI (Kim et al., 2001; Bakuła et al., 2016). Noteworthy, the inter-subspecies differences have been detected at the protein level. For each of the six (I–VI) *M. kansasii* subspecies, specific matrix-assisted laser desorption-ionization time-of-flight (MALDI TOF) mass spectral profiles have recently been established (Murugaiyan et al., 2018).

Finally, the genetic diversity of *M. kansasii* exists not only between the subspecies but also between strains of the same subspecies. This has been repeatedly evidenced upon AFLP, PFGE, repetitive unit (rep-)PCR profiling (Alcaide et al., 1997; Iinuma et al., 1997; Picardeau et al., 1997; Gaafar et al., 2003; Zhang et al., 2004; Wu et al., 2009; Thomson et al., 2014; Kwenda et al., 2015; Bakuła et al., 2018a) and more recently, by a newly developed typing method, based on the analysis of variable number of tandem repeat (VNTR) loci (Bakuła et al., 2018a).

Across all genetic studies performed so far on *M. kansasii*, a controversy has been growing over the taxonomic rank of the genetic variants of the species, best reflected by their terminology, which includes subspecies, subtypes, and genotypes.

The purpose of this study was to resolve the phylogenetic and taxonomic structure of *M. kansasii* by combining whole genome sequencing with traditional polyphasic classification approaches. The use of a polyphasic strategy, incorporating phylogenetic, biochemical, and chemotaxonomic criteria for resolving the taxonomic identity of mycobacterial species, especially within the NTM group, has been heavily advocated (Saini et al., 2009).

The issue of molecular taxonomy of *M. kansasii* has been addressed in a very recent work by Tagini et al. (2019), which appeared shortly before the completion of our own draft. The present study extends the recently published findings by using a new independent sample and somewhat different methodology, and thus delivers a more refined and complete picture of the *M. kansasii* complex.

## Materials and Methods

### Strains and Culture Conditions

A total of 27 *M. kansasii* strains were used in the study (Table 1). Included in this number were 21 single-patient, epidemiologically unrelated, clinical isolates and 6 environmental isolates. The strains were originally recovered before 2016 from 8 countries, i.e., the Netherlands (*n* = 8), Poland (*n* = 5), the Czech Republic (*n* = 4), Spain (*n* = 2), Belgium (*n* = 1), Germany (*n* = 3), South Korea (*n* = 3), and Italy (*n* = 1). Patients from whom the strains had been collected were classified as having, or not, a pulmonary NTM disease according to the criteria of the American Thoracic Society (ATS) (Griffith et al., 2007). The type strains of *M. kansasii* (ATCC 12478^T^), *M. gastri* (DSM 43505^T^), *M. marinum* (DSM 44344^T^), *M. szulgai* (DSM 44166^T^), *M. conspicuum* (DSM 44136^T^), *M. riyadhense* (DSM 45176^T^). and *M. tuberculosis* H37Rv were also included in the study.

**Table 1:** Epidemiological, microbiological, and clinical characteristics of *M. kansasii* strains under the study.

**No**.	**ID**	**Type^a^**	**Strain no**.	**Collected at^b^**	**Collection date**	**Geographic location**	**Host disease**	**Isolation source^c^**	**PCR-RFLP**			**PCR-sequencing^d^**				**GenBank no**.
									***hsp65***	***rpoB***	***tuf***	***hsp65***	***rpoB***	***tuf***	**ITS**	
1	5MK	I^*^	NLA001000521	RUMC	2010	Nijmegen, Netherlands	NTM disease	BAL	I	I	I	ND	I	ND	95%	MWKY01
2	6MK	I	ATCC25221	FB	1968	Borstel, Germany	NTM disease	sputum	I	I	I	I	I	ND	I	CP019885
3	1MK	I	NLA001000927	RUMC	2010	Nijmegen, Netherlands	NTM disease	sputum	I	I	I	I	I	ND	I	CP019883
4	4MK	I	NLA001000449	RUMC	2010	Nijmegen, Netherlands	NTM disease	sputum	I	I	I	I	I	I	I	CP019884
5	10MK	I	6200	WMU	2008	Warsaw, Poland	NTM disease	sputum	I	I	I	I	I	ND	I	CP019886
6	9MK	I	7728	WMU	2009	Warsaw, Poland	NTM disease	BW	I	I	I	I	I	ND	I	CP019888
7	11MK	I	7744	WMU	2009	Warsaw, Poland	NTM disease	BW	I	I	I	I	I	ND	I	CP019887
8	5JD	I	1010001495	RUMC	ND	Czech Republic	NA	water	I	I	I	I	I	ND	I	LWCL01
9	K4	I	K4	SMC	2008	Ulsan, South Korea	NTM disease	sputum	HaeIII: I BstEII: II	I	I	96%	I	I	I	NKQW01
10	K14	I^**^	K14	SMC	2011	Seoul, South Korea	NTM disease	sputum	HaeIII: I BstEII: II	II	II	96%	95%	97%	95%	NKQY01
11	K19	I^**^	K19	SMC	2012	Seoul, South Korea	none	sputum	HaeIII: I BstEII: II	II	II	96%	95%	97%	95%	NKQX01
12	12MK	II	2193	WMU	2011	Warsaw, Poland	none	BW	II	II	II	97%	95%	ND	95%	MWQA01
13	3MK	II	NLA001001128	RUMC	2010	Nijmegen, the Netherlands	none	BAL	II	II	II	96%	95%	ND	96%	MWKX01
14	7MK	II	B11073207	RUMC	2011	Nijmegen, Netherlands	none	sputum	II	II	II	97%	95%	ND	95%	MWKZ01
15	8MK	II	B11063838	RUMC	2011	Nijmegen, Netherlands	none	BAL	II	II	II	96%	95%	97%	96%	MWKV01
16	3B/6JD	II	1010001469	RUMC	ND	Italy	NA	water	II	II	II	97%	95%	ND	95%	LWCM01
17	H47	II	H47	HB	1999	Bilbao, Spain	NTM disease	sputum	II	II	II	ND	ND	ND	ND	NKRA01
18	H48	II	H48	HB	1999	Bilbao, Spain	none	sputum	II	II	II	ND	ND	ND	ND	NKQZ01
19	14_15	III	14_15	IPH	2015	Šumperk, Czech Rep.	none	sputum	III	III	III	ND	ND	ND	ND	NKRB01
20	174_15	III	174_15	IPH	2015	Karviná, Czech Rep.	NTM disease	sputum	III	III	III	ND	ND	ND	ND	NKRD01
21	3JD	III	1010001468	RUMC	ND	Belgium	NA	soil	III	III	III	ND	96%	95%	91%	LWCJ01
22	2JD	IV	1010001458	RUMC	ND	Germany	NA	tap water	III	III	III	97%	93%	96%	93%	LWCI01
23	241/15	IV	241/15	IPH	2015	Karviná, Czech Rep.	none	sputum	IV	IV	IV	ND	ND	ND	ND	NKRE01
24	1JD	V	1010001454	RUMC	ND	Germany	NA	tap water	V	V	V	95%	96%	96%	92%	LWCH01
25	4JD	V	1010001493	RUMC	ND	The Netherlands	NA	water	V	V	V	95%	97%	96%	93%	LWCK01
26	49_15	V	49_11	HID	2011	Warsaw, Poland	none	BW	V	V	V	ND	ND	ND	ND	NKRC01
27	2MK	VI	NLA001001166	RUMC	2010	Nijmegen, Netherlands	none	sputum	VI	VI	VI	96%	92%	94%	91%	MWKW01

a According to the WGS-based (MiSI method) grouping; M. kansasii strains NLA001000521, K14, and K19 had originally been described as types I/II

(*) and IIb

(**) *, based on the PCR-RFLP/PCR-sequencing analysis of the hsp65, rpoB, and/or tuf genes*.

b *RUMC, Radboud University Medical Center, Nijmegen, the Netherlands; FB, Forschungszentrum Borstel, Germany; WMU, Department of Internal Medicine, Pulmonology, and Allergology, Warsaw Medical University, Warsaw, Poland; SMC, Department of Medicine, Samsung Medical Center, Sungkyunkwan University School of Medicine, Seoul, South Korea; HB, Hospital de Basurto, Bilbao, Spain; IPH, Institute of Public Health in Ostrava, Czech Republic; HID, Hospital of Infectious Diseases in Warsaw, Poland*.

c *BAL, bronchoalveolar lavage; BW, bronchial washing*.

d *M. kansasii type was established based on at least 99% similarity of a query sequence with the respective one of the M. kansasii ATCC12478 (type I) reference strain. For other than type I strains, similarity of the query sequence with the respective sequence of the M. kansasii ATCC12478 reference strain was indicated*.

*NA, not applicable; ND, no data*.

All strains were maintained as frozen stocks and cultured on Löwenstein-Jensen or Middlebrook 7H10 agar (Becton-Dickinson, Franklin Lakes, USA) medium, supplemented with oleic acid, albumin, dextrose, and catalase, and incubated at either 30 or 37°C.

### Species Identification and Genotyping

The strains were identified as *M. kansasii* by using high pressure liquid chromatography (HPLC) of cell wall mycolic acids, in accordance with the Centers for Disease Control and Prevention (CDC) guidelines (Butler et al., 1996) and by the GenoType Mycobacterium CM/AS assay (Hain Lifescience, Nehren, Germany), according to the manufacturer's instructions. For genotypic identification, total DNA was purified from solid bacterial cultures with a standard extraction method described previously (Santos et al., 1992). Genotyping of *M. kansasii* strains was performed by PCR-RFLP analysis of the *hsp65, rpoB*, and *tuf* genes, as reported elsewhere (Telenti et al., 1993; Kim et al., 2001; Bakuła et al., 2016).

### Biochemical Profiling

The strains were evaluated for a panel of biochemical characteristics by conventional laboratory procedures (CLSI; Clinical and Laboratory Standards Institute, 2011). These comprised tests for niacin accumulation, nitrate and tellurite reduction, Tween 80 hydrolysis (10 days), catalase (thermostable and semi-quantitative), β-glucosidase, arylsulfatase (3 and 14 days), urease (5 days), and pyrazynamidase. Inhibition tests of tolerance to thiphene-2-carboxylic acid hydrazide (TCH), 5% sodium chloride, and acidic (pH 5.5) conditions were also carried out. In addition, cultural features, including colony morphology, photochromogenicity, the ability to grow on MacConkey agar without crystal violet, and at different temperatures (25, 35, and 45°C) were assessed. For each assay, each strain was tested in triplicate. Only if at least two replications produced identical results, the test was considered complete.

### Drug Susceptibility Testing

To test antimicrobial susceptibility, minimal inhibitory concentrations (MICs) for 13 drugs, including isoniazid (INH), rifampicin (RMP), streptomycin (STR), ethambutol (EMB), clarithromycin (CLR), amikacin (AMK), rifabutin (RFB), moxifloxacin (MFX), ciprofloxacin (CFX), co-trimoxazole (SXT), linezolid (LZD), doxycycline (DOX), and ethionamide (ETO), were determined by the microdilution method, using the Sensititre® SLOMYCO plates (TREK Diagnostic Systems, Cleveland, USA), following the Clinical and Laboratory Standards Institute (CLSI) recommendations (CLSI; Clinical and Laboratory Standards Institute, 2011).

### Genome Sequencing and Assembly

For the whole-genome sequencing, chromosomal DNA from all 27 *M. kansasii* strains under the study was extracted by mechanical cell (100 mg pellet) disruption by using zirconia ceramic beads in a FastPrep-24 instrument (MP Biomedicals, Valiant Co., Yantai, China) and further extracted chemically followed with a DNAzol® reagent (Invitrogen, Carlsbad, USA).

DNA concentration, its purity and integrity was determined using a Qubit high-sensitivity (HS) assay kit (ThermoFisher, Waltham, USA).

Paired-end libraries were prepared from 1 ng of high-quality genomic DNA with the Nextera XT DNA sample preparation kit according to the manufacturer's instructions (Illumina Inc., San Diego, USA). The libraries were sequenced on a HiSeq 2500 or a NextSeq 500 instrument (Illumina, San Diego, USA) at a read length of 2 × 150 bp. The quality of reads before and after pre-processing was assessed using FastQC (v0.11.5) (Andrews, 2010). The raw reads were trimmed with TrimGalore ver. 0.43 (http://www.bioinformatics.babraham.ac.uk/projects/trim_galore/), and *de novo* assembled with the SPAdes Genome Assembler ver. 3.10.0 (Nurk et al., 2013). Scaffold-level assembly was tested using Quast (QUality ASsesment Tool) (Gurevich et al., 2013) to generate basic genome statistics.

In addition to genomes of 27 *M. kansasii* strains, sequenced in this study, genomes of other 53 *Mycobacterium* sp. strains were analyzed. The genomic sequences of these strains were retrieved from the GenBank database (http://www.ncbi.nlm.nih.gov/genbank/) and their appropriate accession numbers were provided in Supplementary Table 6.

### Genomic Data Availability

The assembled genomes were deposited under NCBI Bio-Project accession numbers: PRJNA374853 and PRJNA317047. The genomes were deposited in the GenBank under accession numbers provided in Table 1 and Supplementary Table 6.

### Annotation and Comparative Genome- and Gene-Scale Analyses

Gene identification and annotation were achieved using the DFAST pipeline v1.0.5 with default settings (Tanizawa et al., 2017).

The genomic relatedness between the strains analyzed was assessed using the MiSI (Microbial Species Identifier) method, based on a combination of genome-wide average nucleotide identity (gANI) and alignment fraction (AF) of orthologous genes (Varghese et al., 2015). The gANI and AF values of 96.5 and 0.6, respectively, were assumed as cutoffs for species delimitation.

As a second method to evaluate the genomic relatedness, the Genome-to-Genome Distance Calculator (GGDC at http://ggdc.dsmz.de) was used. This algorithm was designed to replace standard DNA–DNA hybridization (DDH) by calculating DNA-DNA relatedness (Meier-Kolthoff et al., 2013a). The genome-to-genome distance (GGD) value of 0.0258 was identified as a maximum threshold to assign a genome pair to the same species.

The pairwise ANI and AF values were determined from using ANIcalculator (v1.0) (Yoon et al., 2017).

Full-length sequences of the 16S rRNA (1537 bp; MH794239.1), *hsp65* (664 bp; NC_000962.3:528608–530230), *rpoB* (3439 bp; C_000962.3:759807–763325), *tuf* (1191 bp; NC_000962.3:784821–786011) genes, and the 16S-23S rRNA intergenic spacer region (ITS) (358 bp; AL123456.3:1473359–1473716) were extracted from the whole-genome sequence of the *M. tuberculosis* reference strain H37Rv, using CLC Genomic Workbench 10 (Qiagen, Valencia, USA), and were used, each, as a reference for alignments with the respective sequences of other mycobacterial species. Sequences from the same loci of the draft genomes analyzed were identified using the blastn algorithm. Due to a significant variation of the sought sequences, whenever necessary, they were extracted manually or downloaded from the NCBI database (https://www.ncbi.nlm.nih.gov/).

### Identification of RD1-14 Genes

To establish the presence of genes within 14 regions of deletions RD1-14, DIFFIND software with -c 0.7 (sequence identity threshold) and -s2 0.5 (length difference cut-off) parameters was used (Marciniak et al., 2017). Identification of RD1-14 genes was based on the alignment of amino acid sequences of predicted genes from the analyzed genomes to the reference database. As references were used amino acid sequences produced by RD1-14 genes from the *M. tuberculosis* reference strain H37Rv (Accession no.: NC_000962.3) and listed by Brosch et al. (2002).

### Phylogenetic Analysis

Core-genome single-ortholog tree was built as described previously (Tagini et al., 2019). Briefly, OrthoFinder (ver. 2.3.4) and MAFFT (ver. 7.310) were used to create core-genome concatemers for the analyzed genomes (Katoh and Standley, 2013; Emms and Kelly, 2019), which were submitted to FastTree (version 2.1.11 Double precision) for maximum-likelihood core-genome phylogeny calculations (Price et al., 2010). The tree was midpoint rooted in FigTree version 1.4.4 (https://github.com/rambaut/figtree).

For single-gene phylogenies, the respective sequences of each genetic locus were subjected to multiple alignment in MEGA X software (ClustalW algorithm) (Kumar et al., 2018). The resulting fragments were further trimmed to remove unnecessary gaps or regions, to a final length of 1,537, 644, 3,439, 1,180, and 277 bp for 16S RNA *hsp65, rpoB, tuf*, and 16S-23S rRNA ITS region, respectively. The so prepared sequences were used for evolutionary distance calculation according to the Jukes-Cantor model (Jukes and Cantor, 1969). Phylogenetic trees for each target locus sequences were built using the neighbor-joining method and midpoint rooted with the MEGA X software (Saitou and Nei, 1987). Tree topologies were evaluated by bootstrap analysis based on 1,000 replications (Felsenstein, 1985).

Furthermore, a concatenated tree was constructed based on the 16sRNA, *rpoB*, and *hsp65* gene sequences combined. Sequence concatenation was performed with Sequence Matrix 1.8 software (Vaidya et al., 2011), further corrected in Geneious Prime (Kearse et al., 2012), and plotted in MEGA X. Sequence similarity matrices were generated using Bioedit ver. 7.0.5 (IDENTIFY matrice) (Hall, 1999).

## Results and Discussion

Sequencing of 27 *M kansasii* genomes yielded a mean coverage of 66.4x per genome. The average number of contigs per genome was 129 (±229) corresponding to an average N50 score of 4933 kb (±2,605 kb).

The genome sizes and GC contents ranged from 5.6 to 6.6 Mbp (avg. 6.2 Mbp ± 0.3 Mbp) and from 65.86 to 66.38 (avg. 66.14 ± 0.10), respectively. These values were consistent with the data reported for previously assembled *M. kansasii* genomes (Table 2).

**Table 2:** Genome features of *M. kansasii* strains under the study.

**No**.	**ID**	**Type^a^**	**Strain no**.	**Sequencing platform**	**Genome size (bp)**	**Coverage**	**GenBank no**.
1	5MK	I*	NLA001000521	MiSeq	6 649 816	44.6	MWKY01
2	6MK	I	ATCC25221	MiSeq	6 462 452	36	CP019885
3	1MK	I	NLA001000927	MiSeq	6 421 275	35	CP019883
4	4MK	I	NLA001000449	MiSeq	6 440 784	39	CP019884
5	10MK	I	6200	MiSeq	6 421 364	39	CP019886
6	9MK	I	7728	MiSeq	6 463 923	33	CP019888
7	11MK	I	7744	MiSeq	6 434 062	45.8	CP019887
8	5JD	I	1010001495	NextSeq 500	6 358 240	28	LWCL01
9	K4	I	K4	NextSeq 500	6 503 804	58	NKQW01
10	K14	I**	K14	NextSeq 500	6 640 863	60	NKQY01
11	K19	I**	K19	NextSeq 500	6 652 978	60	NKQX01
12	12MK	II	2193	MiSeq	6 254 980	33	MWQA01
13	3MK	II	NLA001001128	MiSeq	6 251 123	46	MWKX01
14	7MK	II	B11073207	MiSeq	6 123 476	34.6	MWKZ01
15	8MK	II	B11063838	MiSeq	6 126 434	44	MWKV01
16	3B/6JD	II	1010001469	NextSeq 500	6 266 032	34	LWCM01
17	H47	II	H47	NextSeq 500	6 238 170	53	NKRA01
18	H48	II	H48	NextSeq 500	6 217 650	29	NKQZ01
19	14_15	III	14_15	HiSeq2500	6 363 138	293	NKRB01
20	174_15	III	174_15	HiSeq2500	6 342 319	173	NKRD01
21	3JD	III	1010001468	NextSeq 500	6 141 835	24	LWCJ01
22	2JD	IV	1010001458	NextSeq 500	6 027 332	37	LWCI01
23	241/15	IV	241/15	HiSeq2500	6 093 288	147	NKRE01
24	1JD	V	1010001454	NextSeq 500	6 171 688	25	LWCH01
25	4JD	V	1010001493	NextSeq 500	5 627 124	29	LWCK01
26	49_11	V	49_11	HiSeq2500	6 634 420	280	NKRC01
27	2MK	VI	NLA001001166	MiSeq	6 443 486	33	MWKW01

a According to the WGS-based (MiSI method) grouping; M. kansasii strains NLA001000521, K14, and K19 had originally been described as types I/II (*) and IIb (**), based on the PCR-RFLP/PCR-sequencing analysis of the hsp65, rpoB, and/or tuf genes.

The whole-genome-level relatedness among the 27 *M. kansasii* strains under the study was assessed with three species identification-relevant parameters, namely the alignment fraction (AF) of orthologous genes, the average nucleotide identity (ANI), and the genome-to-genome distance (GGD) (Tables 3 and 4). The AF/ANI metrics were also computed for genomic sequences of another 32 *M. kansasii* strains and 24 *Mycobacterium* sp. strains, representing 5 NTM species and 5 *M. tuberculosis* complex species, all extracted from NCBI databases (Supplementary Table 6). Whereas, the GGD analysis was performed on 69 genomes in total, including 59 *M. kansasii* genomes and 10 genomes representing single NTM (other than *M. kansasii*) and *M. tuberculosis* complex species (Supplementary Table 6).

**Table 3:** Pairwise alignment fractions (**A**) and average nucleotide identities (**B**) for *M. kansasii* subtypes and outgroup species.

**Subtype/group^a^**	***M. kansasii* I**	***M. kansasii* II+**	***M. kansasii* II**	***M. persicum***	***M. kansasii* III**	***M. kansasii* IV**	***M. kansasii* V**	***M. kansasii* VI**	**MTBC**	***M. tuberculosis***	***M. africanum***	***M. bovis***	***M. caprae***	***M. microti***	***M. conspicuum***	***M. gastri***	***M. marinum***	***M. riyadhense***	***M. szulgai***
**A**																			
*M. kansasii* I	**0.69–1.0**																		
*M. kansasii* II+	0.65–0.9	**0.89–0.99**																	
*M. kansasii* II	0.65–0.89	0.89–0.99	**0.91–0.99**																
*M. persicum*	0.65–0.9	0.89–0.99	0.89–0.99	**0.92–0.99**															
*M. kansasii* III	0.64–0.87	0.75–0.86	0.75–0.85	0.76–0.86	**0.85–0.99**														
*M. kansasii* IV	0.63–0.85	0.78–0.84	0.78–0.84	0.79–0.84	0.74–0.84	**0.97**													
*M. kansasii* V	0.61–0.89	0.75–0.88	0.75–0.88	0.77–0.87	0.73–0.89	0.81–0.88	**0.85–0.98**												
*M. kansasii* VI	0.64–0.87	0.8–0.86	0.8–0.85	0.8–0.86	0.74–0.84	0.76–0.82	0.75–0.86	**0.9–0.98**											
MTBC	0.4–0.75	0.48–0.75	0.48–0.75	0.49–0.74	0.47–0.74	0.52–0.75	0.51–0.74	0.49–0.74	**0.9–1.0**										
*M. tuberculosis*	0.41–0.73	0.5–0.73	0.5–0.73	0.51–0.72	0.48–0.73	0.54–0.73	0.53–0.72	0.5–0.72	0.91–0.99	**0.95–0.99**									
*M. africanum*	0.4–0.74	0.5–0.74	0.5–0.74	0.51–0.73	0.48–0.73	0.54–0.74	0.53–0.73	0.49–0.73	0.92–0.99	0.93–0.97	**0.96–0.99**								
*M. bovis*	0.4–0.75	0.49–0.75	0.49–0.75	0.5–0.74	0.47–0.74	0.53–0.75	0.52–0.74	0.49–0.74	0.9–1.0	0.91–0.97	0.93–0.98	**0.96–1.0**							
*M. caprae*	0.4–0.73	0.5–0.73	0.5–0.73	0.51–0.72	0.48–0.73	0.54–0.73	0.53–0.73	0.49–0.72	0.92–0.97	0.93–0.97	0.95–0.97	0.94–0.97	–^*^						
*M. microti*	0.4–0.7	0.48–0.7	0.48–0.7	0.49–0.7	0.47–0.7	0.52–0.7	0.51–0.7	0.49–0.69	0.9–0.94	0.91–0.93	0.92–0.93	0.9–0.94	0.92–0.92	–^*^					
*M. conspicuum*	0.45–0.6	0.55–0.59	0.55–0.58	0.55–0.59	0.53–0.59	0.56–0.6	0.54–0.62	0.56–0.59	0.43–0.65	0.45–0.64	0.44–0.64	0.44–0.65	0.44–0.63	0.43–0.62	–^*^				
*M. gastri*	0.6–0.84	0.74–0.83	0.74–0.83	0.75–0.82	0.72–0.84	0.82–0.86	0.79–0.86	0.73–0.81	0.54–0.75	0.57–0.73	0.56–0.74	0.56–0.75	0.56–0.73	0.54–0.7	0.54–0.6	–^*^			
*M. marinum*	0.5–0.68	0.63–0.68	0.64–0.67	0.63–0.68	0.6–0.68	0.63–0.68	0.62–0.72	0.63–0.66	0.48–0.74	0.5–0.73	0.5–0.73	0.49–0.74	0.5–0.73	0.48–0.7	0.55–0.55	0.62–0.7	–^*^		
*M. riyadhense*	0.5–0.66	0.61–0.65	0.61–0.65	0.62–0.65	0.58–0.66	0.64–0.65	0.63–0.7	0.61–0.64	0.53–0.77	0.55–0.75	0.55–0.76	0.54–0.77	0.55–0.75	0.53–0.72	0.55–0.59	0.65–0.68	0.59–0.63	–^*^	
*M. szulgai*	0.5–0.69	0.62–0.68	0.63–0.67	0.62–0.68	0.59–0.68	0.62–0.68	0.62–0.73	0.63–0.67	0.47–0.73	0.49–0.71	0.49–0.72	0.48–0.73	0.49–0.72	0.47–0.68	0.59–0.6	0.61–0.7	0.62–0.62	0.66–0.71	–^*^
**B**																			
*M. kansasii* I	**98.82–100.0**																		
*M. kansasii* II+	93.2–94.22	**99.66–99.99**																	
*M. kansasii* II	93.2–94.22	99.66–99.99	**99.66–99.98**																
*M. persicum*	93.25–94.16	99.68–99.99	99.68–99.99	**99.7–99.99**															
*M. kansasii* III	93.23–93.78	92.71–92.91	92.76–92.87	92.71–92.91	**99.61–100.0**														
*M. kansasii* IV	92.9–93.3	93.8–93.93	93.8–93.89	93.81–93.93	92.62–92.81	**99.87–99.9**													
*M. kansasii* V	93.96–94.22	94.72–94.91	94.72–94.91	94.73–94.91	93.61–93.77	94.38–94.51	**98.9–99.99**												
*M. kansasii* VI	90.48–90.88	88.55–88.73	88.55–88.71	88.58–88.73	90.4–90.53	88.82–88.96	89.18–89.34	**99.66–99.94**											
MTBC	81.52–81.79	81.6–81.79	81.6–81.79	81.64–81.78	81.57–81.81	81.85–81.98	81.88–82.04	81.04–81.22	**99.83–100.0**										
*M. tuberculosis*	81.52–81.72	81.6–81.71	81.6–81.7	81.64–81.71	81.57–81.74	81.85–81.97	81.88–82.0	81.04–81.15	99.83–99.96	**99.88–99.96**									
*M. africanum*	81.56–81.74	81.66–81.78	81.66–81.77	81.69–81.78	81.65–81.77	81.89–81.97	81.9–82.01	81.07–81.18	99.87–99.98	99.87–99.94	**99.92–99.98**								
*M. bovis*	81.56–81.76	81.66–81.79	81.66–81.79	81.69–81.78	81.66–81.81	81.91–81.98	81.93–82.04	81.07–81.22	99.87–100.0	99.87–99.94	99.9–99.95	**99.95–100.0**							
*M. caprae*	81.57–81.71	81.64–81.73	81.64–81.7	81.68–81.73	81.65–81.74	81.91–81.95	81.9–81.99	81.08–81.14	99.83–99.96	99.83–99.89	99.88–99.93	99.94–99.96	–^*^						
*M. microti*	81.65–81.79	81.65–81.74	81.65–81.71	81.7–81.74	81.68–81.8	81.94–81.97	81.9–82.01	81.06–81.18	99.89–99.94	99.89–99.91	99.92–99.94	99.9–99.93	99.9–99.9	–^*^					
*M. conspicuum*	80.79–80.97	80.88–81.09	80.91–81.06	80.88–81.09	80.81–81.0	81.05–81.12	81.06–81.19	80.39–80.47	81.23–81.33	81.26–81.33	81.23–81.29	81.26–81.32	81.3–81.32	81.27–81.28	–^*^				
*M. gastri*	92.13–92.39	92.89–93.04	92.94–93.03	92.89–93.04	92.1–92.27	95.15–95.18	93.64–93.76	88.73–88.79	81.85–81.95	81.88–81.91	81.85–81.89	81.86–81.91	81.9–81.91	81.93–81.95	81.12–81.14	–^*^			
*M. marinum*	81.37–81.53	81.29–81.48	81.29–81.46	81.32–81.48	81.33–81.57	81.64–81.67	81.71–81.79	80.96–81.01	80.34–80.47	80.34–80.43	80.35–80.43	80.39–80.47	80.39–80.41	80.41–80.43	79.81–79.84	81.61–81.62	–^*^		
*M. riyadhense*	81.63–81.83	81.63–81.78	81.63–81.72	81.66–81.78	81.66–81.86	81.99–82.04	82.03–82.12	81.19–81.25	82.5–82.61	82.54–82.59	82.5–82.56	82.55–82.61	82.54–82.55	82.55–82.56	81.2–81.23	82.06–82.07	80.39–80.41	–^*^	
*M. szulgai*	81.06–81.35	81.18–81.49	81.18–81.49	81.18–81.25	81.11–81.45	81.34–81.43	81.37–81.46	80.69–80.77	81.13–81.25	81.16–81.22	81.13–81.19	81.18–81.25	81.17–81.2	81.21–81.22	80.82–80.82	81.41–81.42	80.06–80.06	83.19–83.21	–^*^

a *Each M. kansasii subtype was represented by a group of 2–31 strains (genomes). The M. kansasii II group included 7 strains (2193, NLA001001128, B11073207, B11063838, 1010001469, H47, and H48); the M. persicum group included 4 strains [AFPC-000227 (T) (Shahraki et al., 2017), MK4, MK15, and MK42 (Tagini et al., 2019)]; the M. kansasii II+ group incorporated strains of both M. kansasii II and M. persicum groups (11 strains in total). The M. kansasii groups III, V, and VI included strains of respective genotypes collected for this study as well as strains which had elsewhere been designated as M. pseudokansasii (MK21, MK35, and MK142), M. innocens (MK13), and M. attenuatum (MK41, MK136, MK191), accordingly (Tagini et al., 2019). In addition, genomes of six M. tuberculosis, five M. africanum, five M. bovis, and single M. caprae, M. microti, M. conspicuum, M. gastri, M. marinum, M. riyadhense, and M. szulgai strains were analyzed. The MTBC contained genomes of all the aforesaid M. tuberculosis, M. africanum, M. bovis, M. caprae, and M. microti strains (18 strains in total). See Supplementary Table 6*.

* *The values were not given, since only one genome sequence per species was analyzed; AF/ANI values calculated within the groups of strains (genomes) are indicated in bold. The AF and gANI values of 0.6 and 96.5, respectively, were identified as minimum thresholds to assign a genome pair to the same species*.

**Table 4:** Genome-to-genome distance (GGD) of *M. kansasii* subtypes and other mycobacterial outgroup species.

**Subtype/group^a^**	***M. kansasii* I**	***M. kansasii* II+**	***M. kansasii* II**	***M. persicum***	***M. kansasii* III**	***M. kansasii* IV**	***M. kansasii* V**	***M. kansasii* VI**	**MTBC**	***M. tuberculosis***	***M. africanum***	***M. bovis***	***M. caprae***	***M. microti***	***M. conspicuum***	***M. gastri***	***M. marinum***	***M. riyadhense***	***M. szulgai***
*M. kansasii* I	**0.0–0.0138**																		
*M. kansasii* II+	0.0657–0.077	**0.0002–0.0045**																	
*M. kansasii* II	0.0657–0.077	0.0003–0.0045	**0.0004–0.0045**																
*M. persicum*	0.0665–0.0766	0.0002–0.0044	0.0003–0.0044	**0.0002–0.004**															
*M. kansasii* III	0.0707–0.0755	0.0802–0.0818	0.0804–0.0818	0.0802–0.0817	**0.0001–0.006**														
*M. kansasii* IV	0.0751–0.0796	0.0699–0.0707	0.0701–0.0707	0.0699–0.0706	0.0826–0.0837	**0.0025**													
*M. kansasii* V	0.0656–0.0688	0.0601–0.0614	0.0601–0.0613	0.0602–0.0614	0.0715–0.0732	0.0647–0.0654	**0.0006–0.014**												
*M. kansasii* VI	0.1006–0.1038	0.123–0.124	0.123–0.124	0.123–0.1239	0.106–0.1068	0.1214–0.1221	0.1185–0.1197	**0.0011–0.0045**											
MTBC	0.1861–0.1889	0.1864–0.1879	0.1864–0.1879	0.1868–0.1875	0.1866–0.1878	0.1865–0.1872	0.1851–0.1865	0.1903–0.1914	**0.0016–0.0042**										
*M. tuberculosis*	0.1867–0.1886	0.1866–0.1876	0.1866–0.1876	0.187–0.1874	0.1868–0.1876	0.1869–0.187	0.1854–0.1864	0.1912–0.1914	0.0025–0.0042	–^*^									
*M. africanum*	0.1861–0.1882	0.1864–0.1875	0.1864–0.1875	0.1868–0.1871	0.1866–0.1874	0.1865–0.1866	0.1851–0.1859	0.1903–0.1905	0.0022–0.0041	0.0041	–^*^								
*M. bovis*	0.1867–0.1886	0.1866–0.1876	0.1866–0.1876	0.1869–0.1873	0.1868–0.1875	0.1868–0.1869	0.1854–0.1862	0.1909–0.1911	0.0016–0.0026	0.0025	0.0022	–^*^							
*M. caprae*	0.1867–0.1884	0.1866–0.1877	0.1866–0.1877	0.187–0.1874	0.1867–0.1875	0.1869–0.1869	0.1852–0.1862	0.1912–0.1913	0.0016–0.0038	0.0038	0.0035	0.0016	–^*^						
*M. microti*	0.1869–0.1889	0.1867–0.1879	0.1867–0.1879	0.187–0.1875	0.187–0.1878	0.1871–0.1872	0.1855–0.1865	0.1911–0.1911	0.0026–0.0042	0.0042	0.0029	0.0026	0.0038	–^*^					
*M. conspicuum*	0.1925–0.1947	0.1915–0.1933	0.1915–0.1932	0.1916–0.1933	0.1925–0.1939	0.1921–0.1922	0.1909–0.192	0.1957–0.1971	0.1934–0.194	0.1937	0.1934	0.194	0.194	0.1939	–^*^				
*M. gastri*	0.0842–0.0866	0.0788–0.0797	0.0791–0.0797	0.0788–0.0797	0.087–0.0883	0.0563–0.0567	0.0721–0.0728	0.1228–0.1231	0.1856–0.1863	0.1863	0.1856	0.1861	0.1862	0.1863	0.1928	–^*^			
*M. marinum*	0.1876–0.1905	0.1886–0.1892	0.1886–0.1892	0.1886–0.1891	0.1893–0.1902	0.1891–0.1891	0.1872–0.1881	0.194–0.1942	0.1974–0.1982	0.1981	0.1974	0.198	0.1982	0.198	0.2027	0.1888	–^*^		
*M. riyadhense*	0.185–0.1881	0.1872–0.1878	0.1872–0.1878	0.1872–0.1876	0.1865–0.1874	0.1849–0.1852	0.1843–0.1857	0.1904–0.1911	0.1822–0.1828	0.1828	0.1826	0.1822	0.1826	0.1828	0.1919	0.1851	0.1981	–^*^	
*M. szulgai*	0.1886–0.1934	0.1887–0.1931	0.1887–0.1931	0.1924–0.1931	0.1871–0.1922	0.1914–0.1914	0.1904–0.1915	0.1939–0.1943	0.1929–0.1935	0.1932	0.1934	0.193	0.1935	0.1929	0.195	0.1909	0.2024	0.1726	–^*^

a *Each M. kansasii subtype was represented by a group of 2–31 strains (genomes). The M. kansasii II group included 7 strains (2193, NLA001001128, B11073207, B11063838, 1010001469, H47, and H48); the M. persicum group included 4 strains [AFPC−000227 (T) (Shahraki et al., 2017), MK4, MK15, and MK42 (Tagini et al., 2019)]; the M. kansasii II+ group incorporated strains of both M. kansasii II and M. persicum groups (11 strains in total). The M. kansasii groups III, V, and VI included strains of respective genotypes collected for this study as well as strains which had elsewhere been designated as M. pseudokansasii (MK21, MK35, and MK142), M. innocens (MK13), and M. attenuatum (MK41, MK136, MK191), accordingly (Tagini et al., 2019). In addition, genomes of single M. tuberculosis, M. africanum, M. bovis, M. caprae, M. microti, M. conspicuum, M. gastri, M. marinum, M. riyadhense, and M. szulgai strains were analyzed. The MTBC contained genomes of all the aforesaid M. tuberculosis, M. africanum, M. bovis, M. caprae, and M. microti strains (5 strains in total). See Supplementary Table 6*.

* *The values were not given, since only one genome sequence per species was analyzed. GGDs calculated within the groups of strains (genomes) are indicated in bold. The GGD values lower than 0.0258 were used to assign a genome pair to the same species*.

The AF values for all *M. kansasii* strains ranged between 0.6 and 1.0. The lowest AF value recorded for two strains, members of the same *M. kansasii* genotype was 0.69 (type I, range 0.69–1.0). Within all other types, the AF values were 0.85 or higher.

All *M. kansasii* genotypes showed AF values equal to or below 0.75 with strains of other *Mycobacterium* species, except for *M. gastri* and *M. persicum*, which yielded AF values as high as 0.86 with *M. kansasii* type IV and V (range, 0.79–0.86) or 0.99 with *M. kansasii* type II (range, 0.89–0.99), respectively (Table 3A).

Pairwise ANI values for any *M. kansasii* strains affiliated with different types never exceeded the 95–96% threshold (range, 88.6–94.9%), commonly used as a boundary of species delineation (Richter and Rosselló-Móra, 2009; Varghese et al., 2015). Whereas, the ANI values for strains of the same *M. kansasii* genotype were always higher than 98.8%. The ANI values between *M. kansasii* and other *Mycobacterium* species were all below 95% (range, 80.4–94.9%) except between *M. kansasii* genotype II and *M. persicum* (range, 99.7–99.9%) and between *M. kansasii* genotype IV and *M. gastri* (95.2%) (Table 3B).

**Figure 1:**
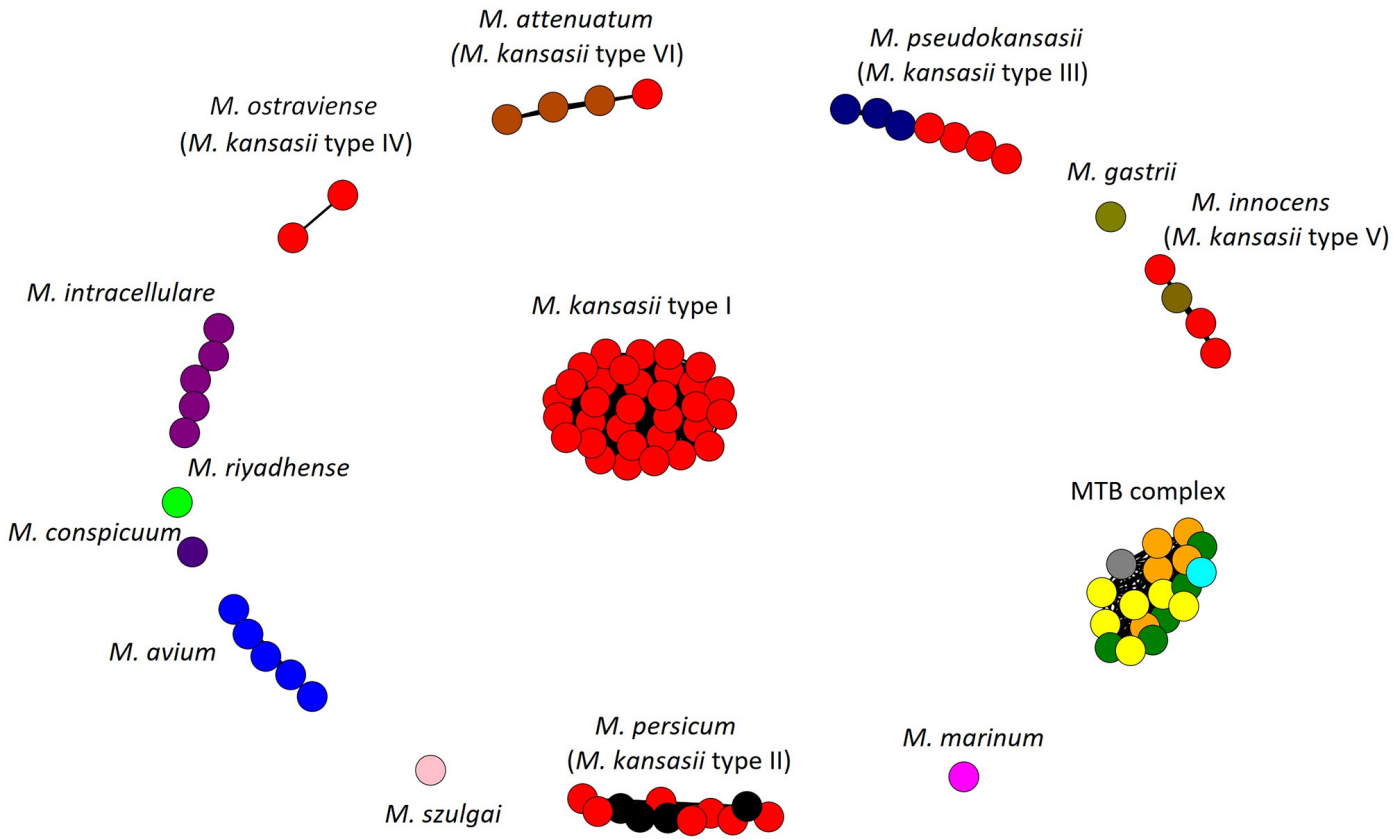
Application of the Microbial Species Identifier (MiSI) method in classifying *Mycobacterium kansasii* subtype I-VI strains. Separation of different species into different cliques is depicted. Each color represents a species, while each circle—a genome of that species. Within cliques corresponding to *M. persicum* (formerly subtype II), *M. pseudokansasii* (III), *M. innocens* (V), and *M. attenuatum* (VI), genomes, which had been analyzed previously and first assigned to newly proposed species are shown in other than red color. Within the *M. tuberculosis* (MTB) complex clique, genomes of *M. tuberculosis, M. africanum, M. bovis, M. caprae*, and *M. microti* are depicted in yellow, orange, green, blue, and gray, respectively.

The AF and ANI metrics were also analyzed combinatorially, using the Microbial Species Identifier (MiSI) algorithm. The MiSI method sorts the analyzed genomes into species-like taxa or cliques, based on the AF and ANI species-level cut-off values set at 0.6 and 96.5%, respectively (Varghese et al., 2015). For a total of 83 mycobacterial genomes studied, 15 different cliques were configured. The results were pictorially summarized in Figure 1. The genomes of 59 *M. kansasii* strains were clearly divided into six cliques, each containing strains of a distinct *M. kansasii* subtype (I–VI) only. All the remaining NTM species had their genomes clustered within separate cliques, except that four genomes of strains classified as *M. persicum* were allocated in the *M. kansasii* genotype II clique. The genomes of 19 strains, representing five *M. tuberculosis* complex species were accommodated in a single cluster (clique).

The results of the AF/ANI calculations were fully corroborated by the GGD analysis, which serves as an *in silico* equivalent of the laboratory-based DNA-DNA hybridization (DDH) (Auch et al., 2010; Meier-Kolthoff et al., 2013b). Here, all strains contained within the *M. kansasii* genotypes shared enough sequence similarity to be considered as separate species (Table 4). The intra-genotype GGD values ranged from 0.0 to 0.0138, and thus fell under the recommended cut-off value of ≤0.0258, corresponding to a 70% DDH cut-off, for species demarcation (Meier-Kolthoff et al., 2013b). Much higher were the GGD values between strains representing different *M. kansasii* genotypes (range, 0.0601–0.124) or between any *M. kansasii* and any other *Mycobacterium* species (range, 0.0563–0.1971). The only exception was when comparing genomes of *M. kansasii* type II to any of *M. persicum*, for which the GGD values were between 0.0003 and 0.0044 (Table 4).

**Figure 2:**
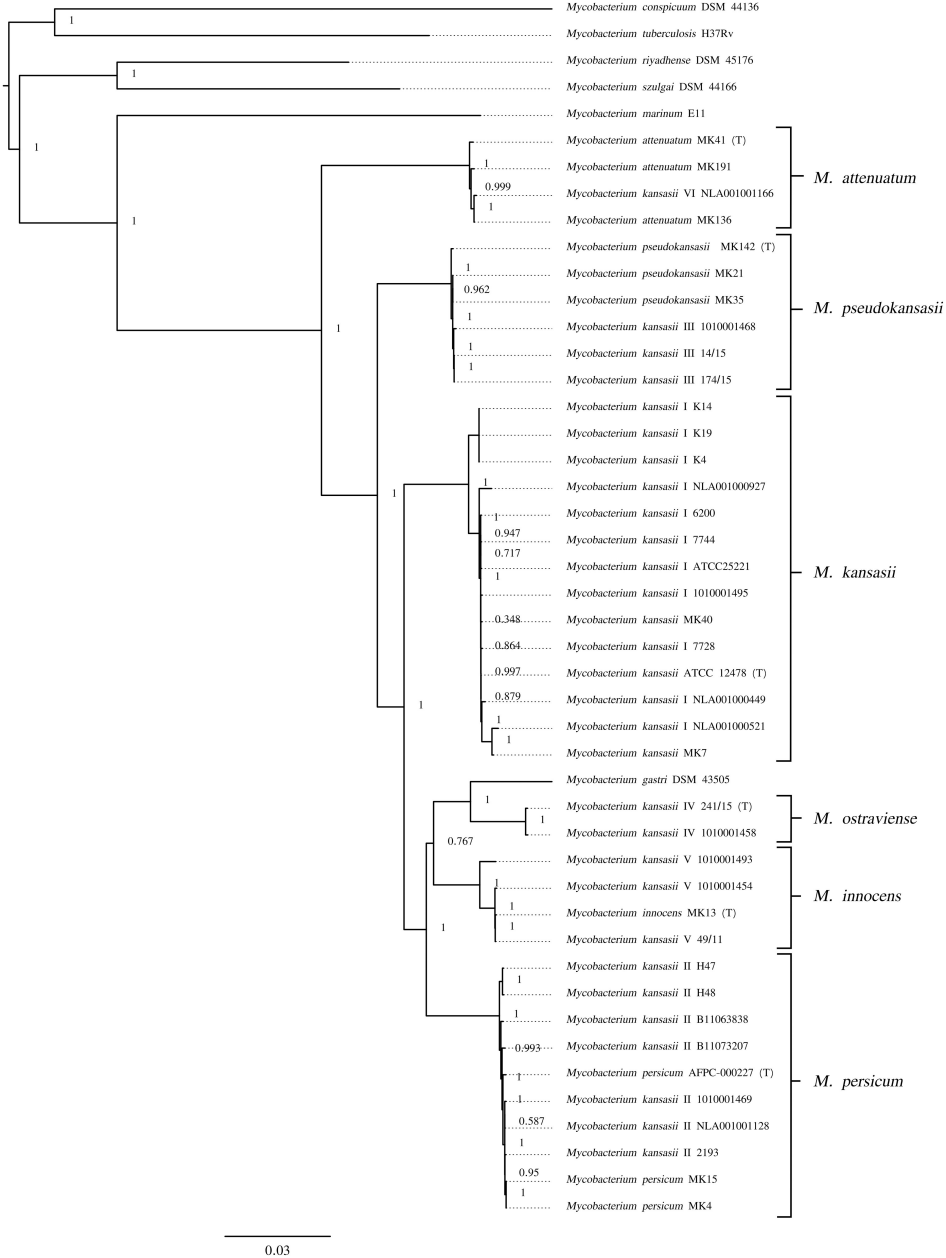
Maximum-likelihood phylogenetic tree based on the amino acid alignment of concatenated single-copy orthologous genes. Bar, number of amino acid substitutions per amino acid site. Node supports were computed using the Shimodaira-Hasegawa test.

Another whole genome-level approach to clarify the taxonomic relationships between the six *M. kansasii* subtypes was the core-genome phylogenetic analysis. A dendrogram based on the concatenated 615,565-amino-acid sequences from 1,752 single-copy orthologous genes clearly separated all *M. kansasii* subtypes (Figure 2). All strains belonging to the same subtype formed distinct clades, supported by high bootstrap values (76.7–100%). Noteworthy, strains of *M. persicum* located in the same clade as the *M. kansasii* type II strains, whereas *M. gastri* branched sisterly to *M. kansasii* type IV. The topology of the tree supports the separation of *M. kansasii* subtypes, as distinct species, with *M. kansasii* type II being conspecific with *M. persicum*.

The taxonomic position of *M. kansasii* genotypes was also examined using phylogenetic inferences from five genetically conserved loci, including the canonical 16S rRNA gene, the ITS region, and three protein-coding genes, namely *hsp65, tuf*, and *rpoB*, all being widely applied as molecular markers for the classification of mycobacteria (Tortoli, 2014). Multi-alignment and phylogenetic analyses for each of the five loci was conducted on the sequences of all 59 *M. kansasii* strains and single strains of *M. tuberculosis* and five other NTM species (Supplementary Table 6).

Pairwise alignments of the 1,537-bp sequences of 16S rRNA gene from *M. kansasii* strains showed that they were highly similar or identical (99–100% sequence similarity), both within and between the subtypes (Supplementary Table 1). At the same time, strains of *M. kansasii* shared 98–98.6% similarity with *M. tuberculosis* and more than 98% similarity with other NTM species. The 16S rRNA gene sequences were identical between *M. persicum* and *M. kansasii* type II, and between *M. gastri* and *M. kansasii* types I and IV.

Comparisons of the 277-bp ITS sequences from *M. kansasii* strains showed at most 85 and 98% similarities with the corresponding sequences from *M. tuberculosis* and other NTM species, respectively. Only sequences from *M. kansasii* type II and *M. persicum* were almost identical, sharing 99.2–100% similarity (Supplementary Table 2). The ITS sequence similarities within and between different *M. kansasii* subtypes fell under relatively wide ranges, i.e., 92.1–100% and 81.2–100%, respectively. The highest inter-subtype similarity values (>95%) were observed between three *M. kansasii* type I strains (K14, K19, and NLA00100521) and strains of *M. kansasii* type II. The type II-specific ITS sequences of those three type I strains accounted for the high intra-type heterogeneity (92.1–100% sequence similarity).

Sequence analysis of the 644-bp *hsp65* gene fragments showed similarities of 90.3–92.3% between *M. kansasii* and *M. tuberculosis*, and 90.9–97.9% between *M. kansasii* and other NTM species, except *M. persicum* which shared 99.5–100% similarity with *M. kansasii* type II (Supplementary Table 3). Alignments of the *hsp65* gene sequences from members of different *M. kansasii* subtypes yielded similarities of <98%. The only exception were three strains (K4, K14, and K19) of *M. kansasii* type I sharing up to 99.8% similarity with *M. kansasii* type II strains.

The results of the partial *tuf* (1,180 bp) gene analysis were similar to those obtained with the *hsp65* gene (Supplementary Table 4). The *tuf* gene sequence similarities between *M. kansasii* and *M. tuberculosis* were 93.4% at most, while those between *M. kansasii* and other NTM species were always below 98%, except that sequences of *M. kansasii* type II and *M. persicum* were identical. The similarity indexes calculated for *M. kansasii* of different subtypes did not exceed 98.3%, except that two strains of type I (K14 and K19) had the same *tuf* sequences as *M. kansasii* type II strains.

Finally, alignments of the partial *rpoB* (3,439 bp) gene sequences found all *M. kansasii* strains to share <89% sequence similarity with *M. tuberculosis* and <96% similarity with NTM species, but again not *M. persicum*, whose sequences were identical or nearly identical with those of *M. kansasii* type II (Supplementary Table 5). The level of the *rpoB* gene sequence similarity between members of different *M. kansasii* subtypes was consistently below 98%, excluding two type I strains (K14 and K19), which displayed high similarity or identity with type II strains.

**Figure 3:**
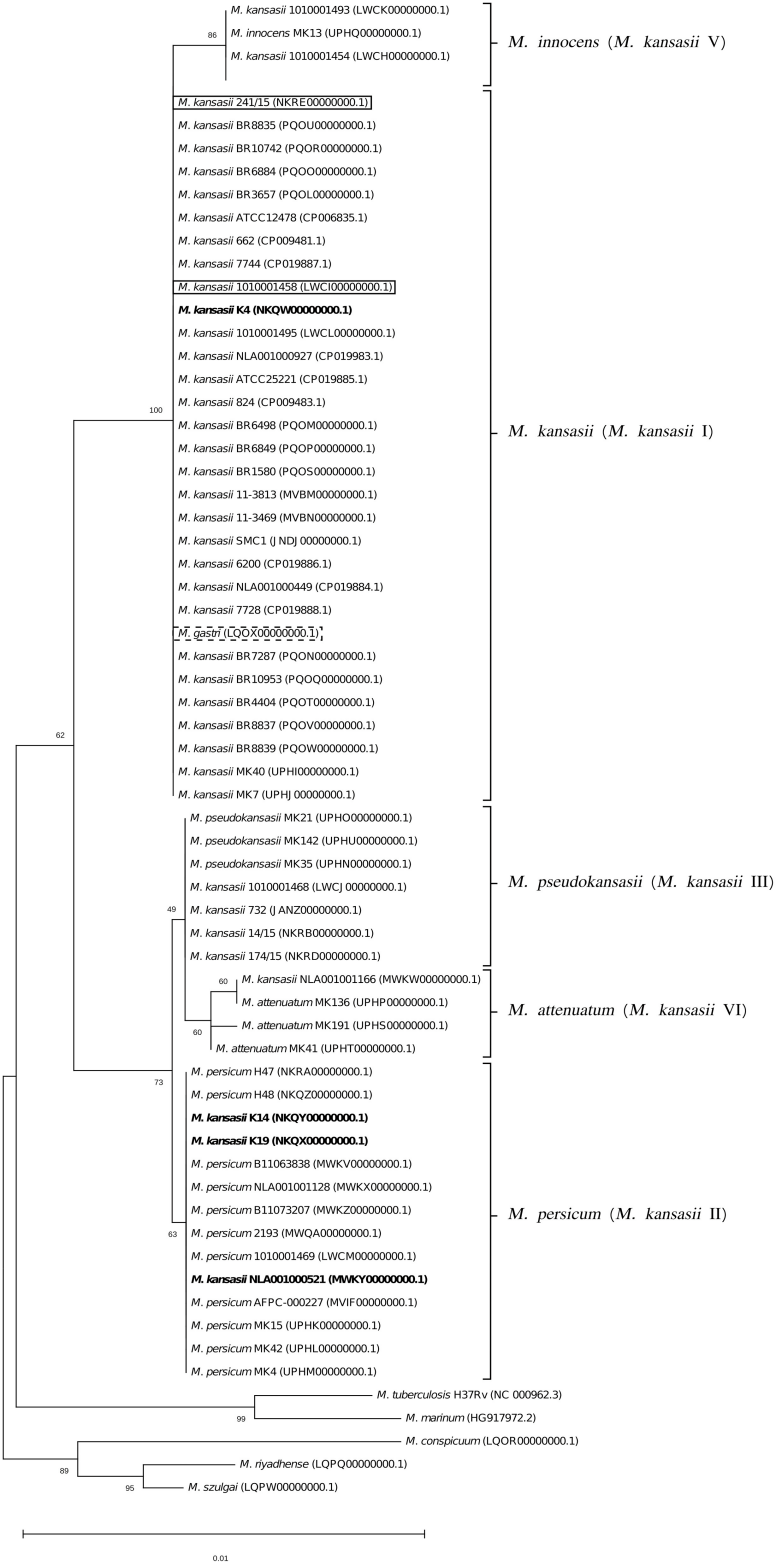
Phylogenetic tree based on 16S rRNA gene sequences, constructed using the Neighbor-Joining method. The bootstrap values were calculated from 1,000 replications. Bootstrap values are given at nodes. Strains of *M. kansasii* type IV (241/15, 1010001458) and *M. gastri* are shown boxed in solid and dashed lines, respectively. Strains of *M. kansasii* type I, as per WGS-based analysis, that clustered either within type I or II, upon single-gene or concatenated gene phylogenies are shown in bold. GenBank accession numbers for the sequences are parenthesized. Bar, 0.01 substitutions per nucleotide position.

**Figure 4:**
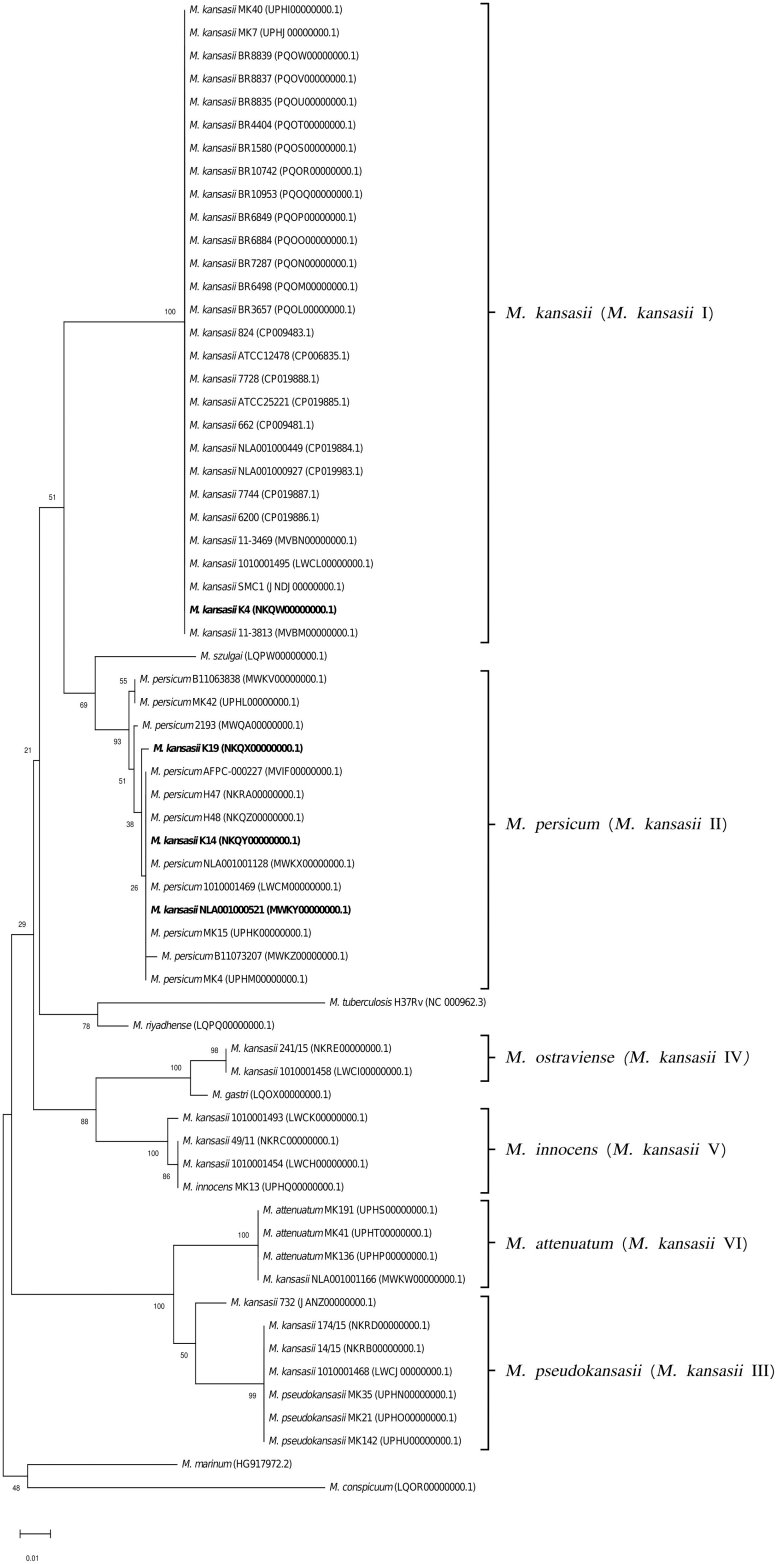
Phylogenetic tree based on ITS sequences constructed using the Neighbor-Joining method. The bootstrap values were calculated from 1,000 replications. Bootstrap values are given at nodes. Strains of *M. kansasii* type I, as per WGS-based analysis, that clustered either within type I or II, upon single-gene or concatenated gene phylogenies are shown in bold. GenBank accession numbers for the sequences are parenthesized. Bar, 0.01 substitutions per nucleotide position.

**Figure 5:**
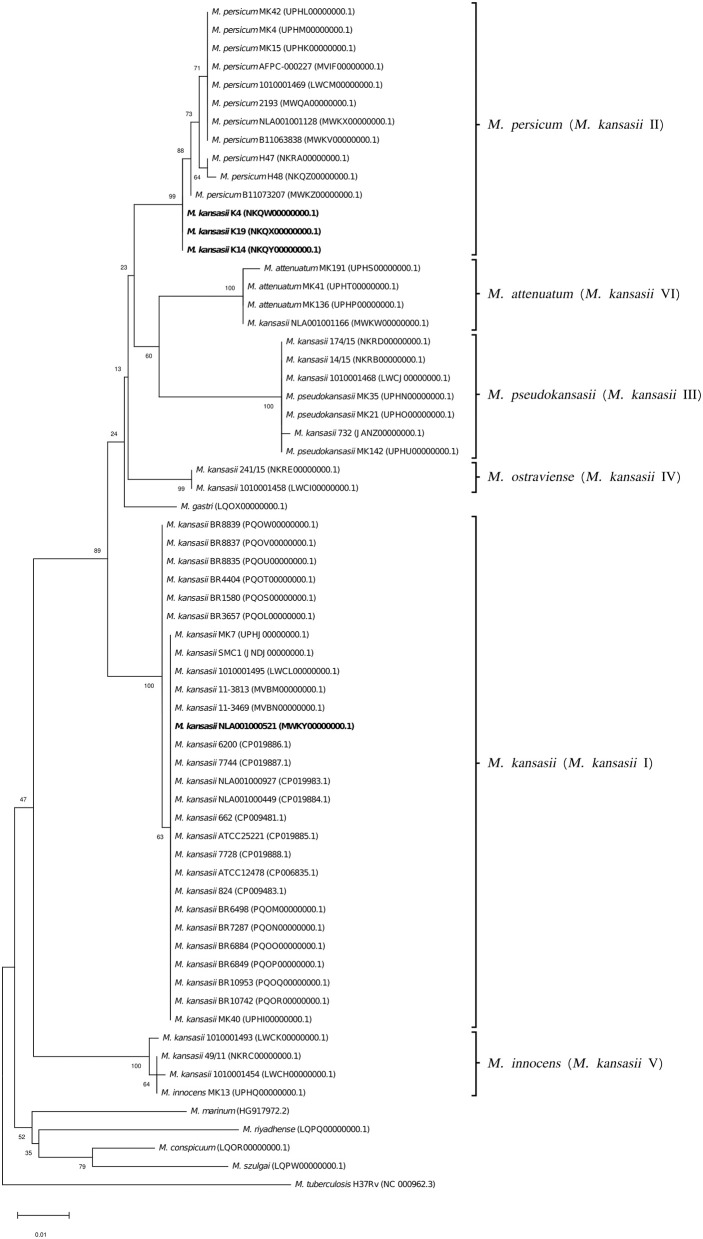
Phylogenetic tree based on *hsp65* gene sequences constructed using the Neighbor-Joining method. The bootstrap values were calculated from 1,000 replications. Bootstrap values are given at nodes. Strains of *M. kansasii* type I, as per WGS-based analysis, that clustered either within type I or II, upon single-gene or concatenated gene phylogenies are shown in bold. GenBank accession numbers for the sequences are parenthesized. Bar, 0.01 substitutions per nucleotide position.

**Figure 6:**
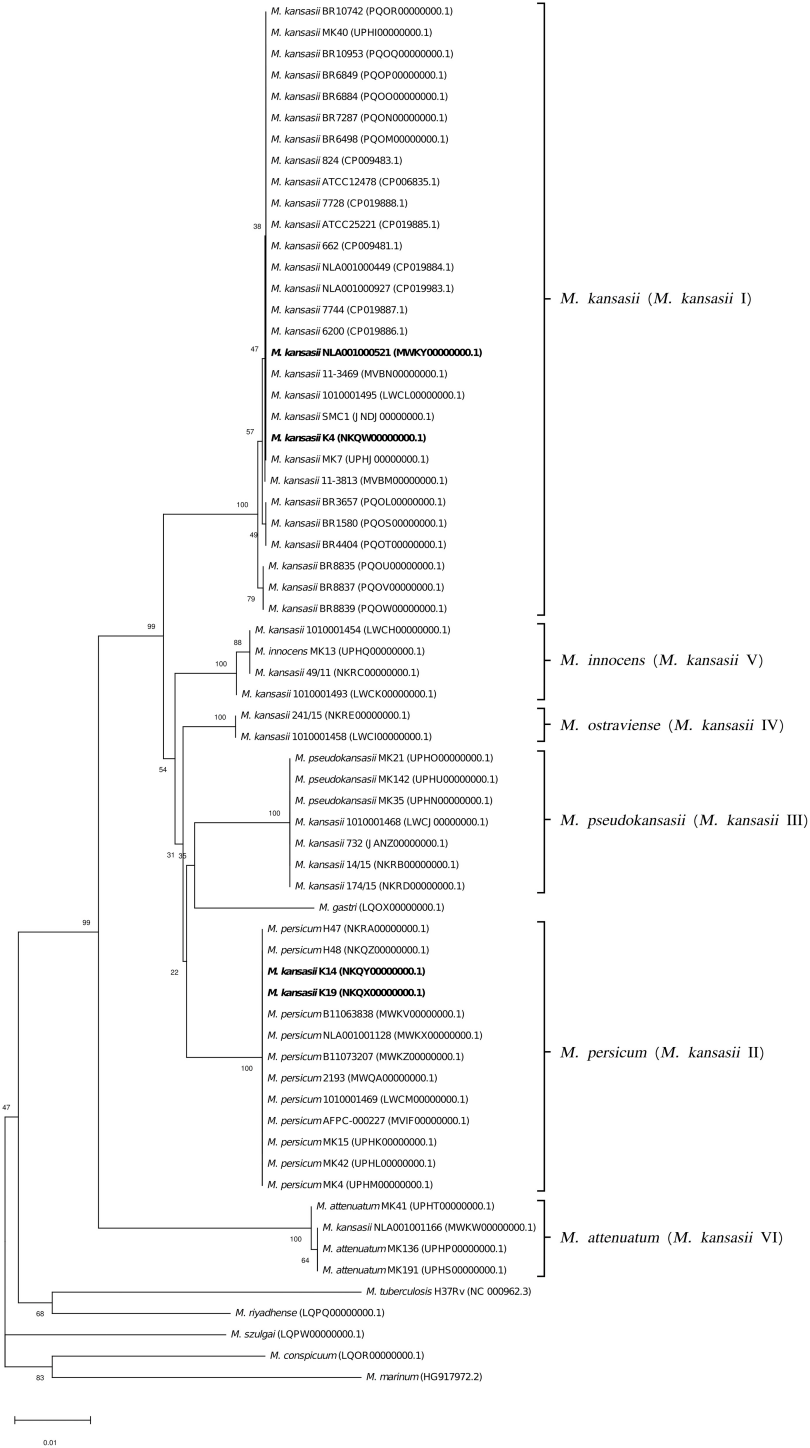
Phylogenetic tree based on *tuf* gene sequences constructed using the Neighbor-Joining method. The bootstrap values were calculated from 1,000 replications. Bootstrap values are given at nodes. Strains of *M. kansasii* type I, as per WGS-based analysis, that clustered either within type I or II, upon single-gene or concatenated gene phylogenies are shown in bold. GenBank accession numbers for the sequences are parenthesized. Bar, 0.01 substitutions per nucleotide position.

**Figure 7:**
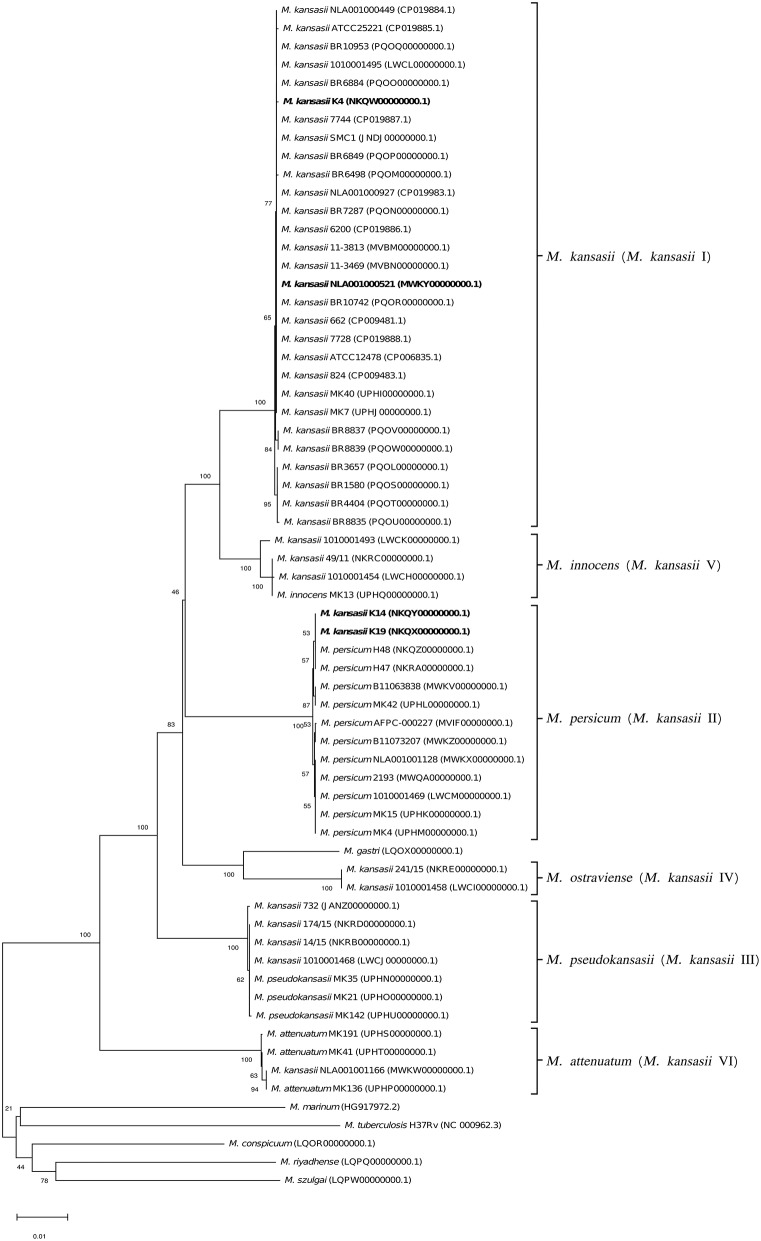
Phylogenetic tree based on *rpoB* sequences constructed using the Neighbor-Joining method. The bootstrap values were calculated from 1,000 replications. Bootstrap values are given at nodes. Strains of *M. kansasii* type I, as per WGS-based analysis, that clustered either within type I or II, upon single-gene or concatenated gene phylogenies are shown in bold. GenBank accession numbers for the sequences are parenthesized. Bar, 0.01 substitutions per nucleotide position.

**Figure 8:**
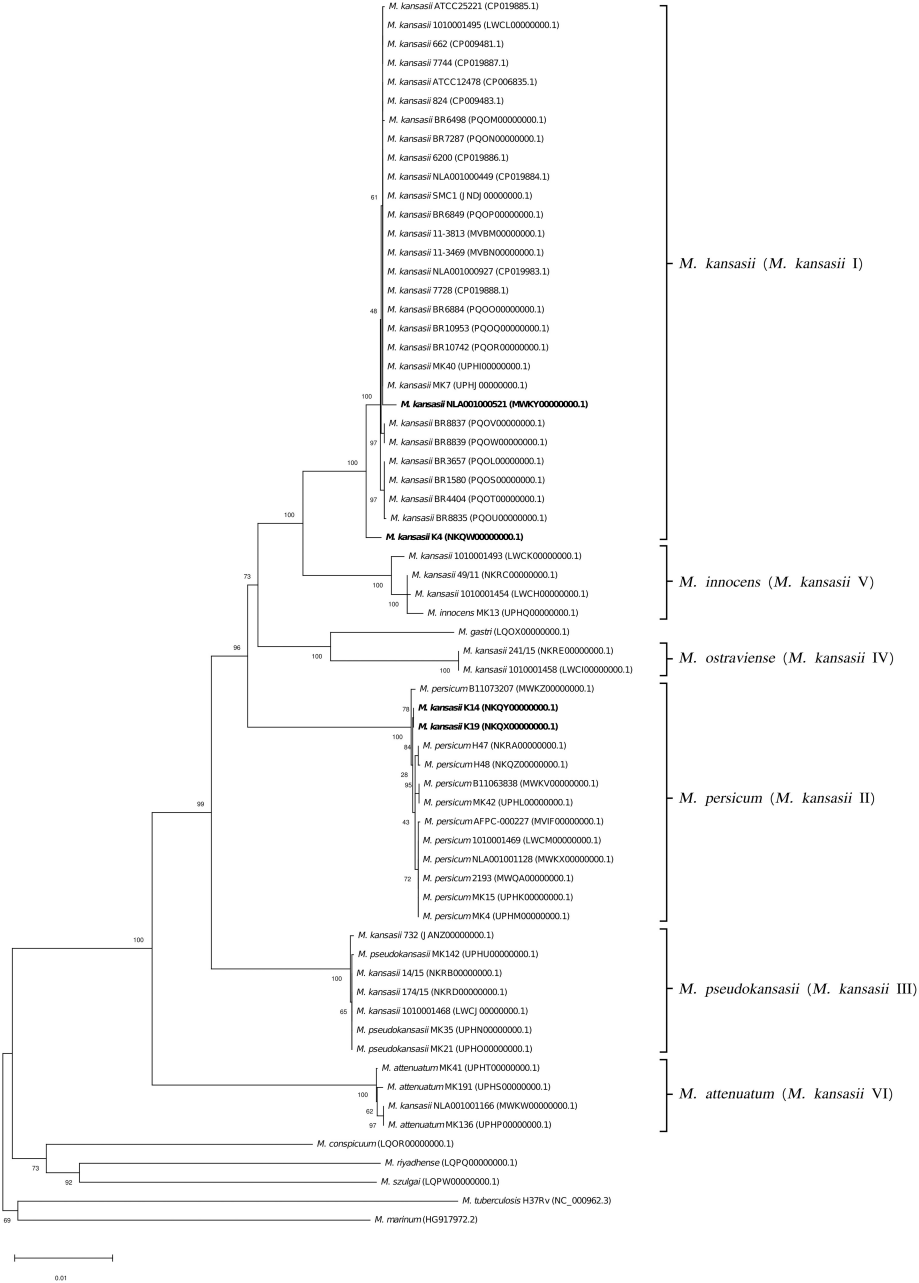
Phylogenetic tree based on concatenated 16S rRNA, *hsp65*, and *rpoB* gene sequences constructed using the Neighbor-Joining method. The bootstrap values were calculated from 1,000 replications. Bootstrap values are given at nodes. Strains of *M. kansasii* type I, as per WGS-based analysis, that clustered either within type I or II, upon single-gene or concatenated gene phylogenies are shown in bold. GenBank accession numbers for the sequences are parenthesized. Bar, 0.01 substitutions per nucleotide position.

To better illustrate the phylogenetic relatedness of *M. kansasii* subtypes, phylogenetic trees inferred from five individual loci were constructed (Figures 3–7). A separate tree was created using the concatenated 16S rRNA, *hsp65*, and *rpoB* genes (Figure 8), since such an approach is known to increase considerably discrimination and robustness of the dendrogram analysis (Devulder et al., 2005). In all but one dendrograms, all *M. kansasii* strains could be spread into six highly supported (bootstrap values ≥ 93%) clusters, according to their subtype affiliation (Figures 4–8). The 16S rRNA gene-based dendrogram was different in that it contained no cluster specific for *M. kansasii* type IV. Two type IV strains clustered together with *M. kansasii* type I strains (Figure 3). Noteworthy, in the same cluster the type strain of *M. gastri* was placed. A feature, which was apparent across all the trees was that strains of *M. kansasii* type II clustered along with *M. persicum*. Moreover, there were four *M. kansasii* type I strains that branched within that cluster. Two of these strains (K14 and K19) were always present in the *M. kansasii* type II-*M. persicum* cluster, whereas another two belonged to that cluster only in the trees based on the ITS region (strain no. NLA00100521), 16S rRNA gene (NLA00100521), and *hsp65* gene (strain no. K4). Having the type I-specific *hsp65* gene sequence (sequevar I) and type II-specific ITS sequence (sequevar II), strain no. NLA00100521 represents the so-called intermediate type I (I/II), considered a transitional form between environmental type II and human-adapted type I (Iwamoto and Saito, 2005). Whereas, strains K14 and K19 can be identified as atypical type II (IIb) due to their type II ITS sequence and unique, yet most similar to type II, *hsp65* sequences (Iwamoto and Saito, 2005). Captivatingly, strain K4 had the same unique *hsp65* gene sequence, with all the other sequences being characteristic of type I. Thus, the strain represents the so far unreported variant of *M. kansasii* type I, which can be tentatively designated as atypical type I (Ib).

Altogether, the results from the genome-wide comparisons demonstrated the six (I–VI) *M. kansasii* subtypes to represent distinct species. This was also the conclusion of the recent study by Tagini et al., who based their results upon ANI and GGD analysis of the genomes of 21 *M. kansasii* strains comprising all six subtypes (Tagini et al., 2019) (Twenty of these strains were used in the present work). Also, single- and multigene phylogenies, highly congruent between this and already published study, were indicative of species-level demarcations between *M. kansasii* subtypes. From these findings, Tagini et al. were first to propose new species designations, namely *M. pseudokansasii, M. innocens*, and *M. attenuatum*, replacing the former types III, V, and VI, respectively. The most prevalent type I was preserved under the ‘*M. kansasii*' designation. Whereas, *M. kansasii* type II was found, as in our study, conspecific with *M. persicum*. Therefore, we share the view of assigning this name to all *M. kansasii* type II strains. In fact, the conspecificity of *M. persicum* and *M. kansasii* type II would have been disclosed upon the original description of the species (*M. persicum*), if the authors had included *M. kansasii* type II in the genome-based comparative analysis (Shahraki et al., 2017).

The two *M. kansasii* type IV strains, analyzed in our study, fully satisfied the genomic criteria for a separate species. This was also implied by our predecessors, but in the absence of any type strain, they could not formally establish the species. Here, we propose a new species name, *Mycobacterium ostraviense* sp. nov., to accommodate *M. kansasii* type IV strains, with a strain no. 241/15, as a type. The description of this new species is given at the end of the article.

The taxonomic status of *M. kansasii* type VII remains an enigma. Neither the strain nor its genomic sequence is available, precluding any relevant phylogenetic analyses. This type was reported only once (Taillard et al., 2003), and given the similarity of its *hsp65* RFLP banding patterns, which served as the only diagnostic means, to those of type III, it is plausible that type VII is a product of misidentification.

Since the mid-1990s, the PCR-RFLP analysis, based on single-copy, orthologous genes (*hsp65, rpoB*, and *tuf*) has been widely used for the identification of a plethora of NTM species, including *M. kansasii* and its subtypes (Alcaide et al., 1997; Devallois et al., 1997; Kim et al., 2001; da Silva Rocha et al., 2002; Santin and Alcaide, 2003; Zhang et al., 2004; Kwenda et al., 2015; Bakuła et al., 2016). However, PCR-RFLP typing may not seldom produce misleading results. Single nucleotide polymorphisms can alter the recognition sites of the restriction enzymes and thus generate either patterns unidentifiable or corresponding to those of other species. Also, sequence analysis, even conducted on a combination of genes, may not resolve the species identity adequately. This is best illustrated in the already discussed strains of atypical type IIb, which despite sharing nearly 99% average nucleotide identity with type I, had their 16S rRNA, *hsp65*, and *rpoB* gene sequences, either individually or concatenated, more closely associated with type II. The type IIb strains would have been considered type II (*M. persicum*), if they had not been inspected at the whole genome-level. Thus, neither PCR-RFLP profiling nor single or multilocus sequencing allows unequivocal identification of *M. persicum*. A definite diagnosis should be supported by the genome-wide analysis. Still, the two type IIb strains, under this study, displayed the type I-specific *hsp65* RFLP patterns, upon digestion with HaeIII (but not with BstEII, which was type II-specific). This feature can be exploited for differentiation between types I and II, if whole-genome sequencing is not affordable. Nevertheless, a new, robust genetic marker allowing for fast and accurate identification of all *M. kansasii*-derived species (former types I-VI), bypassing the need for whole-genome analysis, would be of great benefit. For this, a more in-depth, comparative analysis of the genomes of more strains representing the six *M. kansasii*-derived species, and other NTM species, will have to be undertaken.

There has been a continuing debate on how the differences between *M. kansasii* subtypes translate into pathogenicity. The prevailing view is that only types I and II are true human pathogens, with the latter having been associated with immunodeficiency, and HIV infection in particular, whereas all the remaining types are considered non-pathogenic, and their sporadic isolation from clinical samples has been interpreted as colonization or environmental contamination (Tortoli et al., 1994; Taillard et al., 2003). Indeed, *M. kansasii* type I is the most commonly detected among clinical isolates and the predominant cause of *M. kansasii* disease worldwide (Alcaide et al., 1997; Kim et al., 2001; Gaafar et al., 2003; Santin and Alcaide, 2003; Taillard et al., 2003; Zhang et al., 2004; da Silva Telles et al., 2005; Shitrit et al., 2006; Thomson et al., 2014; Kwenda et al., 2015; Bakuła et al., 2016). Infections attributable to *M. kansasii* type II are much rarer (Taillard et al., 2003; Zhang et al., 2004; Shitrit et al., 2006; Bakuła et al., 2016), and those caused by other types are almost unreported in the literature. Still, types III, IV, and VI have been recognized among clinical isolates (Alcaide et al., 1997; Picardeau et al., 1997; Santin and Alcaide, 2003; Thomson et al., 2014; Kwenda et al., 2015) with types IV and VI implicated in the disease (Santin and Alcaide, 2003; Thomson et al., 2014). Due to the paucity of isolations of *M. kansasii* other than type I, their clinical relevance remains obscure. Some new light on this problem may be shed by the findings of an international, multicenter investigation, currently in progress, on the global distribution of *M. kansasii* subtypes. So far, we have documented nine confirmed cases of *M. kansasii* disease, etiologically linked to either types III, IV, V or VI (Jagielski et al., data unpublished). In this context, the newly proposed species names for types V (*M. innocens*) and VI (*M. attenuatum*) may not reflect the true phenotype of those bacteria.

Searching for the genetic determinants of pathogenicity, a key genomic region associated with *M. tuberculosis* virulence, known as “region of difference 1” (RD1) was interrogated across the genomes of *M. kansasii* subtypes for its functional integrity (i.e., presence of RD1 genes). The RD1 encodes a secretory apparatus (ESX-1 type VII secretion system) responsible for exporting two highly potent antigens and virulence factors—the 6-kDa early secreted antigenic target (ESAT-6) and the 10-kDa culture filtrate protein (CFP-10) (Berthet et al., 1998). These proteins, encoded by the same operon, play a critical role in modulation of the host immune response through inhibition of phagosome maturation, cytosolic translocation of mycobacteria or granuloma formation (MacGurn and Cox, 2007; van der Wel et al., 2007; Volkman et al., 2010). The *esat-6* (*esxA*) and *cfp-10* (*esxB*) genes have also been demonstrated in some NTM species, including *M. kansasii* (types I-V), *M. szulgai, M. marinum*, and *M. riyadhense* (van Ingen et al., 2009). Furthermore, the ESAT-6/CFP-10-mediated translocation of bacilli into the cytosol has been proven to occur in *M. kansasii* type I, but not in *M. kansasii* type V (Houben et al., 2012).

Our analysis showed the presence of six RD1 genes (*rv3871*-5 and *rv3877*), including the ESAT-6 and CFP-10-coding genes and two other genes (*rv3871* and *rv3877*) coding for the essential components of the ESX-1 secretion system, in all types of *M. kansasii* and other NTM species (Table 5). Only single strains of *M. kansasii* type I, *M. marinum*, and *M. szulgai* were devoid of the *rv3872* gene coding for PE35, conjectured to play a role in the regulation of *esxB*/*A* expression (Brodin et al., 2006).

**Table 5:** Distribution of region of difference 1 (RD1) genes among *M. kansasii* strains and other mycobacterial outgroup species.

**No**.	**Species/genotype**	**ID**	**Strain no.^a^**	**Locus**^**b**^									**GenBank no**.
				**Rv3879c**	**Rv3876**	**Rv3878**	**Rv3877**	**Rv3873**	**Rv3871**	**Rv3874**	**Rv3875**	**Rv3872**	
1	*M. kansasii* I	5MK	NLA001000521^D^^*^	–	+	–	+	+	+	+	+	+	MWKY01
2	*M. kansasii* I	6MK	ATCC25221^D^^*^	+	+	–	+	+	+	+	+	+	CP019885
3	*M. kansasii* I	1MK	NLA001000927^D^^*^	–	+	–	+	+	+	+	+	+	CP019883
4	*M. kansasii* I	4MK	NLA001000449^D^^*^	–	+	–	+	+	+	+	+	+	CP019884
5	*M. kansasii* I	10MK	6200^D^^*^	–	+	–	+	+	+	+	+	+	CP019886
6	*M. kansasii* I	9MK	7728^D^^*^	+	+	–	+	+	+	+	+	+	CP019888
7	*M. kansasii* I	11MK	7744^D^^*^	–	+	–	+	+	+	+	+	+	CP019887
8	*M. kansasii* I	5JD	1010001495^E^^*^	–	+	–	+	+	+	+	+	+	LWCL01
9	*M. kansasii* I	K4	K4^D^^*^	+	+	–	+	+	+	+	+	+	NKQW01
10	*M. kansasii* I	K14	K14^D^^*^	+	+	–	+	+	+	+	+	+	NKQY01
11	*M. kansasii* I	K19	K19^N^^*^	+	+	–	+	+	+	+	+	+	NKQX01
12	*M. kansasii* I	–	ATCC12478^D^	–	+	–	+	+	+	+	+	+	CP0006835
13	*M. kansasii* I	–	824^N^^I^	–	+	–	+	+	+	+	+	+	CP009483
14	*M. kansasii* I	–	BR3657^D^	–	+	–	+	+	+	+	+	+	PQOL01
15	*M. kansasii* I	–	BR6849^D^	–	+	–	+	+	+	+	+	+	PQOP01
16	*M. kansasii* I	–	SMC1^E^	–	+	–	+	+	+	+	+	+	JNDJ01
17	*M. kansasii* I	–	BR8837^D^	–	+	–	+	+	+	+	+	+	PQOV01
18	*M. kansasii* I	–	BR6498^D^	+	+	–	+	+	+	+	+	+	PQOM01
19	*M. kansasii* I	–	11_3813^R^	–	+	–	+	+	+	+	+	–	MVBM01
20	*M. kansasii* I	–	BR6884^D^	+	+	–	+	+	+	+	+	+	PQOO01
21	*M. kansasii* I	–	BR10742^D^	+	+	–	+	+	+	+	+	+	PQOR01
22	*M. kansasii* I	–	BR1580^D^	+	+	–	+	+	+	+	+	+	PQOS01
23	*M. kansasii* I	–	BR4404^D^	+	+	–	+	+	+	+	+	+	PQOT01
24	*M. kansasii* I	–	BR8839^D^	+	+	–	+	+	+	+	+	+	PQOW01
25	*M. kansasii* I	–	662^NI^	+	+	–	+	+	+	+	+	+	CP009481
26	*M. kansasii* I	–	BR7287^D^	+	+	–	+	+	+	+	+	+	PQON01
27	*M. kansasii* I	–	BR10953^D^	+	+	–	+	+	+	+	+	+	PQOQ01
28	*M. kansasii* I	–	BR8835^D^	+	+	–	+	+	+	+	+	+	PQOU01
29	*M. kansasii* I	–	11_3469^R^	+	+	–	+	+	+	+	+	+	MVBN01
30	*M. kansasii* I	–	MK40^U^	+	+	–	+	+	+	+	+	+	UPHI01
31	*M. kansasii* I	–	MK7^U^	+	+	–	+	+	+	+	+	+	UPHJ01
32	*M. kansasii* II	12MK	2193^N^^*^	–	–	–	+	+	+	+	+	+	MWQA01
33	*M. kansasii* II	3MK	NLA001001128^N^^*^	–	–	–	+	+	+	+	+	+	MWKX01
34	*M. kansasii* II	7MK	B11073207^N^^*^	–	–	–	+	+	+	+	+	+	MWKZ01
35	*M. kansasii* II	8MK	B11063838^N^^*^	+	–	–	+	+	+	+	+	+	MWKV01
36	*M. kansasii* II	3B/6JD	1010001469^E^^*^	–	–	–	+	+	+	+	+	+	LWCM01
37	*M. kansasii* II	H47	H47^D^^*^	–	–	–	+	+	+	+	+	+	NKRA01
38	*M. kansasii* II	H48	H48^N^^*^	–	–	–	+	+	+	+	+	+	NKQZ01
39	*M. persicum*	–	AFPC−000227^D^	–	–	–	+	+	+	+	+	+	MVIF01
40	*M. persicum*	–	MK15^U^	–	–	–	+	+	+	+	+	+	UPHK01
41	*M. persicum*	–	MK4^U^	–	–	–	+	+	+	+	+	+	UPHM01
42	*M. persicum*	–	MK42^U^	–	–	–	+	+	+	+	+	+	UPHL01
43	*M. kansasii* III	14_15	14_15^N^^*^	+	+	–	+	+	+	+	+	+	NKRB01
44	*M. kansasii* III	174_15	174_15^D^^*^	–	+	–	+	+	+	+	+	+	NKRD01
45	*M. kansasii* III	3JD	1010001468^E^^*^	+	+	–	+	+	+	+	+	+	LWCJ01
46	*M. kansasii* III	–	732^U^	+	+	–	+	+	+	+	+	+	JANZ01
47	*M. pseudokansasii*	–	MK142^D^	–	+	–	+	+	+	+	+	+	UPHU01
48	*M. pseudokansasii*	–	MK21^U^	–	+	–	+	+	+	+	+	+	UPHO01
49	*M. pseudokansasii*	–	MK35^U^	+	+	–	+	+	+	+	+	+	UPHN01
50	*M. kansasii* IV	2JD	1010001458^E^^*^	+	+	–	+	+	+	+	+	+	LWCI01
51	*M. kansasii* IV	241/15	241/15^N^^*^	–	+	–	+	+	+	+	+	+	NKRE01
52	*M. kansasii* V	1JD	1010001454^E^^*^	–	+	–	+	+	+	+	+	+	LWCH01
53	*M. kansasii* V	4JD	1010001493^E^^*^	–	+	–	+	+	+	+	+	+	LWCK01
54	*M. kansasii* V	49_11	49_11^N^^*^	–	+	–	+	+	+	+	+	+	NKRC01
55	*M. innocens*	–	MK13^U^	–	+	–	+	+	+	+	+	+	UPHQ01
56	*M. kansasii* VI	2MK	NLA001001166^N^^*^	–	–	–	+	+	+	+	+	+	MWKW01
57	*M. attenuatum*	–	MK41^N^	–	–	–	+	+	+	+	+	+	UPHT01
58	*M. attenuatum*	–	MK136^U^	–	–	–	+	+	+	+	+	+	UPHP01
59	*M. attenuatum*	–	MK191^U^	+	–	–	+	+	+	+	+	+	UPHS01
60	*M. tuberculosis*	–	Beijing–like	+	+	–	+	+	+	+	+	+	CP017597
61	*M. africanum*	–	GM041182	+	+	+	+	+	+	+	+	+	NC_015758
62	*M. bovis*	–	09_1191	+	+	–	+	+	+	+	+	+	JPFP01
63	*M. caprae*	–	Allgaeu	+	+	+	+	+	+	+	+	+	CP016401
64	*M. microti*	–	12	+	+	+	+	–	–	–	–	–	CP010333
65	*M. conspicuum*	–	DSM44136	ND									LQOR01.1
66	*M. gastri*	–	DSM43505	–	+	–	+	+	+	+	+	+	LQOX01
67	*M. marinum*	–	E11	–	+	–	+	+	+	+	+	–	HG917972
68	*M. riyadhense*	–	DSM45176	–	–	–	+	+	+	+	+	+	LQPQ01
69	*M. szulgai*	–	DSM44166	+	–	–	+	+	+	+	+	–	LQPW01

a The presence of a given gene was marked with a “+” and highlighted in gray; the superscript letters refer to isolation source:

D –clinical strains implicated in NTM disease;

N –clinical strains not implicated in NTM disease;

R –clinical strain from the rhesus macaque (Macaca mulatta);

U –clinical strains with unknown relation to NTM disease;

E –environmental strains. Strains whose genomes were sequenced in this study are marked with an asterisk (*).

The *rv3876* gene was demonstrated in all *M. kansasii* types except for types II (*M. persicum*) and VI (*M. attenuatum*). Neither it was present in *M. szulgai* and *M. riyadhense*. The protein encoded by this gene is an ESX-1 secretion-associated protein EspI. It was shown that inactivation of the *rv3876* gene did not impair secretion of ESAT-6 (Brodin et al., 2006). More recently, however, EspI was found to negatively regulate the ESX-1 secretion system in *M. tuberculosis*, in response to low cellular ATP levels. EspI was thus hypothesized to play a possible role during the latent phase of infection (Zhang et al., 2014).

The *rv3879c* gene, coding for another ESX-1 secretion-associated protein EspK, was variably distributed among *M. kansasii* strains. It was detected in half of the strains of types I and III, while being absent in all but one strains of types II and VI, and all type V strains. EspK seems not to be involved in virulence, since the *rv3879c* homolog deletion mutant of *M. bovis* was not attenuated in the guinea pig model (Inwald et al., 2003). Moreover, similar to *rv3876*, inactivation of the EspK-coding gene did not abolish ESAT-6 secretion (Brodin et al., 2006). Contrastingly, EspK of *M. marinum* was found crucial for the ESX-1-mediated secretion of EspB (Rv3881c), required for virulence and growth in macrophages (McLaughlin et al., 2007). It is thus conceivable that EspK may influence the pathogenicity also in *M. kansasii*.

Collectively, based on the distribution of RD1 genes, neither of the *M. kansasii* types could be categorized as being more or less pathogenic, given that all genes essential for the functioning of the ESX-1 secretion machinery were uniformly present in *M. kansasii* strains, and that the absence of certain genes was reported in both clinically relevant and neutral strains. To explore more in depth the genetic background of virulence in *M. kansasii*, more advanced, functional studies should be performed, with a focus not only on the RD1 genes but several other genes flanking that cluster, which together form the “extended RD1” region. Furthermore, we cannot exclude that the pathogenic and non-pathogenic *M. kansasii* strains differ in terms of expression of the RD1 genes or activity of their proteins. More RD1-targeted experimental investigations are required to validate such scenarios.

Analysis of other than RD1 regions of deletions (RD2-14) did not show any consistent (i.e., shared across all strains of a given species) species-specific pattern of RD genes (Supplementary Data Sheet 2). It was noteworthy, however, that the only two genes found in the RD3 locus were present in either of the two most pathogenic *M. kansasii* types I and II. The *rv1577* gene occurred in more than 90% of *M. kansasii* type I and almost 73% of *M. kansasii* type II. Whereas, the *rv1586* gene was demonstrated exclusively in *M. kansasii* type I, at a frequency of nearly 81%. Likewise, only *M. kansasii* types I and II harbored genes of the RD11 locus. The *rv2651* gene was present in 90 and 73% of *M. kansasii* type I and II, respectively. The *rv2646* was evidenced in slightly more than 60% of *M. kansasii* type I. Interestingly, both RD3 and RD11 represent phage inserts within the *M. tuberculosis* genome, and are thought to generate antigenic variation (Ahmed et al., 2007).

It has been canonically accepted, upon description of new species, to provide a detailed phenotypic characterization. However, in the era of genomic-based bacterial taxonomy, the significance of the phenotype has much eroded and the use of conventional biochemical testing has been increasingly abandoned. The algorithm for routine differential diagnostics of NTM species should obligatorily include only growth rate and pigment production (Tortoli et al., 2017).

For closely-related species, the diagnostic value of phenotyping is virtually negligible, as demonstrated also in this study (Table 6). All strains, irrespective of subtype (species), were almost invariably photochromogenic, niacin-negative, and grew at 25 and 37°C, but not at 45°C, in the presence of 5% (w/v) NaCl or on MacConkey agar without crystal violet. They were all resistant to thiophene-2-carboxylic acid hydrazide (TCH) and presented a strong catalase activity, but none exhibited pyrazynamidase activity or arylsulfatase at 3 days. Some variability was observed when testing for nitrate and tellurite reduction, Tween 80 hydrolysis, and urease activity. The only marked difference between the subtypes (species) was that strains of *M. ostraviense* (formerly *M. kansasii* type IV) were, unlike all other species, unable to reduce nitrate and that their catalase was heat-liable. Whether these features are stable within the species need to be verified on a larger set of strains. Interestingly, both these features are typical for *M. gastri* (Kent and Kubica, 1985), with which *M. ostraviense* shares the highest genetic similarity, as evidenced upon whole-genome analysis. *Mycobacterium gastri*, a casual resident of human stomach and only exceptionally pathogenic (Velayati et al., 2005), is easily distinguishable from all *M. kansasii*-derived species, as it is non-photochromogenic.

**Table 6:** Phenotypic characteristics of *M. kansasii* strains under the study.

**Type/Strain no.^a^**			**Morphology^b^**	**Pigment^c^**	**Growth in/post 7 days at/on^d^:**				**Tolerance of^e^:**		**Niacin**	**Reduction of:**		**Arylsulfatase**		**Tween 80 hydrolysis^f^**	**Catalase^g^**		**Urease^h^**	**PZA^i^**
																	**SQ**	**HS**		
					**25^**°**^C**	**37^**°**^C**	**45^**°**^C**	**MCA**	**TCH**	**NaCl 5%**		**nitrate**	**tellurite**	**3–day**	**14–day**					
	Mycobacterium kansasii^*^		SR	P^99^; S^ <1^; N^ <1^	+	+	–	–^99^	+^99^	–^99^	–^99^	+^99^	–^84^	–^99^	+^55^	+^99^	>45^99^	+^95^	+^97^	–^99^
*M. kansasii*	I (11)	NLA001000521	RY	P	+	+	–	–	+	–	–	+	–	–	3+	+	17	–	–	–
		ATCC25221	RW	N	+	+	–	–	+	–	–	+	–	–	3+	+	74	+	+	–
		NLA001000927	RY	P	+	+	–	–	+	–	–	+	–	–	3+	+	54	+	+	–
		NLA001000449	RY	P	+	+	–	–	+	–	–	+	–	–	3+	+	50	+	–	–
		6200	RY	P	+	+	–	–	+	–	–	+	–	–	4+	+	52	+	–	–
		7728	RY	P	+	+	–	–	+	–	–	+	–	–	3+	+	70	+	–	–
		7744	RY	P	+	+	–	–	+	–	–	+	–	–	2+	+	59	+	–	–
		1010001495	RY	P	+	+	–/+	–	+	–	–	+	–	–	4+	+	65	+	–	–
		K4	RY	P	+	+	–/+	–	+	–	–	+	–	–	3+	+	54	+	–	–
		K14	RY	P	+	+	–	–	+	–	–	+	–	–	3+	+	41	+	+	–
		K19	RY	P	+	+	–	–	+	–	–	+	–	–	3+	+	51	+	+	–
			RY^91^	P^91^	+^100^	+^100^	–^82^	–^100^	+^100^	–^100^	–^100^	+^100^	–^100^	–^100^	≥3+^91^	+^100^	>40^91^	+^91^	–^64^	–^100^
*M. persicum*	II (8)	2193	RY	P	+	+	–	–	+	–	–	–	–	–	3+	–	44	+	–	–
		NLA001001128	RY	P	+	+	–	–	+	–	–	+	+	–	3+	–	40	+	+	–
		B11073207	RY	P	+	+	–	–	+	–	–	+	–	–	3+	–	44	+	+	–
		B11063838	RY	P	+	+	–	–	+	–	–	+	–	–	3+	–	43	+	+	–
		1010001469	RY	P	+	+	–	–	+	–	–	+	–	–	4+	+	18	–	–	–
		H47	RY	P	+	+	–	–	+	–	–	+	+	–	4+	+	45	+	+	–
		H48	RY	P	+	+	–	–	+	–	–	+	–	–	3+	+	47	+	+	–
		AFPC−000227 (T)^**^	RY	P	–	–	–	–	NT	NT	–	+	–	NT	NT	+	>45	+	–	NT
			RY^100^	P^100^	+^88^	+^88^	–^100^	–^100^	+^100^	–^100^	–^100^	+^88^	–^75^	–^100^	≥3+^100^	+^50^	≥40^88^	+^88^	+^63^	–^100^
*M. pseudokansasii*	III (4)	14_15	RY	P	+	+	–	–	+	–	–	+	–	–	3+	+	9	–	+	–
		174_15	RY	P	+	+	–	–	+	–	–	+	–	–	1+	+	8	–	+	–
		1010001468	RY	P	+	+	–	–	+	–	–	+	+	–	3+	+	30	+	–	–
		MK142	RY	P	+	+	–	–	+	–	–	+	–	–	2+	+	45	+	+	–
		MK142 (T)^***^	RY	NT	NT	NT	NT	NT	NT	NT	–	+	NT	NT	NT	+	NT	NT	v	NT
			RY^100^	P^100^	+^100^	+^100^	–^100^	–^100^	+^100^	–^100^	–^100^	+^100^	–^75^	–^100^	≥2+^75^	+^100^	≥30^50^	+^50^	+^60^	–^100^
*M. ostraviense*	IV (2)	1010001458	RY	P	+	+	–	–	+	–	–	–	–	–	1+	–	53	–	–	–
		241/15 (T)	RY	P	+	+	–	–	+	–	–	–	–	–	1+	+	48	–	–	–
			RY^100^	P^100^	+^100^	+^100^	–^100^	–^100^	+^100^	–^100^	–^100^	–^100^	–^100^	–^100^	≥1+^100^	+^50^	>40^100^	–^100^	–^100^	–^100^
*M. innocens*	V (4)	1010001454	RY	P	+	+	–	–	+	–	–	+	–	–	2+	+	78	+	+	–
		1010001493	RW	N	+	+	–	–	+	–	–	+	–	–	1+	+	3	–	–	–
		49_11	RY	P	+	+	–	–	+	–	–	+	–	–	3+	+	43	+	+	–
		MK13 (T)	RY	P	+	+	–	–	+	–	–	+	–	–	1+	–	39	+	+	–
		MK13 (T)^***^	Y	NT	NT	NT	NT	NT	NT	NT	–	v	NT	NT	NT	v	NT	NT	v	NT
			RY^60^	P^80^	+^100^	+^100^	–^100^	–^100^	+^100^	–^100^	–^100^	+^80^	–^100^	–^100^	≥1+^100^	+^60^	>30^75^	+^75^	+^60^	–^100^
*M. attenuatum*	VI (2)	NLA001001166	RY	P	+	+	–	–	+	–	–	+	–	–	3+	–	60	+	+	–
		MK41 (T)	RY	P	+	+	–	–	+	–	–	+	–	–	2+	+	45	+	+	–
		MK41 (T)^***^	Y	NT	NT	NT	NT	NT	NT	NT	v	v	NT	NT	NT	+	NT	NT	v	NT
			RY^67^	P^100^	+^100^	+^100^	–^100^	–^100^	+^100^	–^100^	–^67^	+^67^	–^100^	–^100^	≥2+^100^	+^67^	>40^100^	+^100^	+^67^	–^100^

*All test reactions are given as “+” (positive), “–” (negative) or “v” (variable); NT, not tested. Numbers in superscripts represent percentage of strains reacting as indicated*.

a *According to the WGS-based (MiSI method) grouping. Vertically are given the newly proposed names for each of the M. kansasii subtype; (T), type strain*.

* *Results according to Kent and Kubica (1985); prior to subtype delineation*.

** *Results according to Shahraki et al. (2017)*.

*** *Results according to Tagini et al. (2019)*.

b *Colony morphology: S, smooth; R, rough; Y, yellow/beige; W, white*.

c *Photochromogenicity: N, non-photochromogenic; P, photochromogenic; S, scotochromogenic*.

d *MCA, MacConkey agar without crystal violet*.

e *TCH, thiophene-2-carboxylic acid hydrazide*.

f *After 10 days*.

g *SQ, semi-quantitative catalase [mm]; HS, heat-stable catalase*.

h *after 5 days; v, variable*.

i *PZA, pyrazinamidase activity, after 4 days*.

Finally, drug susceptibility profiles of 30 mycobacterial strains, including type strains of three newly established species, by Tagini et al. (2019), were compared within and between the species (former *M. kansasii* types) (Table 7). Shortly, all strains were susceptible to RIF, RFB, AMK, SXT, MFX, LZD, and CLR (except one CLR-resistant strain of *M. kansasii*). Of these drugs, only RIF and CLR showed interspecies differences in their activity, with the MICs for *M. kansasii* and *M. persicum* slightly higher than for other *M. kansasii*-derived species. More than 80% of strains were resistant to EMB. Among these, were all strains of *M. kansasii* (former type I), *M. persicum* (II), and *M. ostraviense* (IV). Single strains of *M. kansasii, M. pseudokansasii*, and *M. attenuatum* were resistant to CIP. The MICs of STR and DOX varied widely (<0.5–16 mg/L vs. 1–>16 mg/L), yet the highest values (16 vs. >16 mg/L) were observed only for strains of *M. kansasii, M. persicum*, and *M. attenuatum*. The INH and ETO MICs were low, and within relatively narrow ranges (<0.25–2 mg/L vs. <0.3–0.6 mg/L). These findings confirm the key observations from previous studies, on the susceptibility of *M. kansasii* strains to RIF and their high resistance to EMB (da Silva Telles et al., 2005; Wu et al., 2009; Shahraki et al., 2017; Bakuła et al., 2018c). This, confronted with the ATS recommendation of a three-drug (INH-RIF-EMB) regimen for the treatment of *M. kansasii* disease, speaks for exclusion of EMB and its replacement with other potent drug, such as moxifloxacin.

**Table 7:** Drug susceptibility profiles of *M. kansasii* strains under the study.

**Type^a^**		**MIC [mg/L] of^b^**													
		**Strain no**.	**RIF**	**CLR**	**INH**	**EMB**	**STR**	**AMK**	**SXT**	**RFB**	**MXF**	**LZD**	**CIP**	**DOX**	**ETO**
*M. kansasii*	I (11)	NLA001000521	0.25	0.25	2	>16	8	2	<0.12	<0.25	<0.12	2	2	4	<0.3
		ATCC25221	0.25	0.12	2	16	4	2	<0.12	<0.25	<0.12	2	1	4	0.6
		NLA001000927	0.25	0.12	2	16	4	1	<0.12	<0.25	<0.12	1	1	4	0.6
		NLA001000449	0.25	>64	2	>16	8	4	<0.12	<0.25	<0.12	2	2	8	<0.3
		6200	0.25	0.25	2	>16	8	2	0.12	<0.25	<0.12	2	2	16	<0.3
		7728	1	0.5	2	>16	16	8	0.12	<0.25	0.25	4	4	>16	<0.3
		7744	1	0.12	1	>16	4	2	<0.12	<0.25	<0.12	<1	1	4	<0.3
		1010001495	0.25	0.12	2	16	8	2	<0.12	<0.25	<0.12	<1	1	4	<0.3
		K4	0.5	0.25	2	>16	4	2	0.12	<0.25	<0.12	2	1	4	<0.3
		K14	1	0.5	2	>16	8	4	0.12	<0.25	<0.12	2	1	8	<0.3
		K19	0.5	0.25	1	>16	4	2	0.12	<0.25	<0.12	2	0.5	4	<0.3
		MIC50/90	0.25/1	0.25/0.5	2/2	>16/>16	8/8	2/4	<0.12/<0.12	<0.25/<0.25	<0.12/<0.12	2/2	1/2	4/16	<0.3/0.6
		MIC range	0.25-1	0.12->64	1-2	16->16	4-16	1-8	<0.12-0.12	<0.25	<0.12–0.25	<1–4	0.5–4	4–>16	<0.3–0.6
*M. persicum*	II (7)	2193	0.5	0.12	1	16	2	<1	<0.12	<0.25	<0.12	<1	0.25	2	<0.3
		NLA001001128	1	0.25	1	>16	16	8	<0.12	<0.25	<0.12	4	2	8	<0.3
		B11073207	0.25	0.12	1	16	4	2	<0.12	<0.25	<0.12	2	0.5	4	<0.3
		B11063838	1	0.5	1	>16	8	4	<0.12	<0.25	<0.12	2	1	16	<0.3
		1010001469	1	0.12	1	>16	16	2	<0.12	<0.25	<0.12	2	2	8	<0.3
		H47	1	0.5	2	>16	4	2	0.12	<0.25	0.25	2	1	8	0.6
		H48	0.25	1	1	>16	8	8	2	<0.25	<0.12	4	1	8	<0.3
		MIC50/90	1/1	0.25/1	1/2	>16/>16	8/16	2/8	<0.12/2	<0.25/<0.25	<0.12/0.25	2/4	1/2	8/16	<0.3/0.6
		MIC range	0.25–1	0.12–1	1–2	16/>16	2–16	<1–8	<0.12–2	<0.25	<0.12–0.25	<1–4	0.25–2	2–16	<0.3–0.6
*M. pseudokansasii*	III (4)	14_15	<0.12	0.12	1	>16	4	4	0.5	<0.25	<0.12	2	1	1	<0.3
		174_15	0.12	0.12	2	>16	8	2	<0.12	<0.25	<0.12	2	2	4	<0.3
		1010001468	0.5	<0.06	1	16	4	<1	<0.12	<0.25	<0.12	<1	0.5	2	<0.3
		MK142	0.25	0.25	0.5	1	2	<1	<0.12	<0.25	0.25	<1	4	4	<0.3
		MIC50/90	0.12/0.5	0.12/0.25	1/2	16/>16	4/8	<1/4	<0.12/0.5	<0.25/<0.25	<0.12/0.25	<1/2	1/2	2/4	<0.3/<0.3
		MIC range	<0.12–0.5	<0.06–0.25	0.5–2	1–>16	2–8	<1–4	<0.12–0.5	<0.25	<0.12–0.25	<1–2	0.5–4	1–4	<0.3
*M. ostraviense*	IV (2)	1010001458	<0.12	<0.06	1	16	4	2	0.12	<0.25	<0.12	<1	0.5	4	<0.3
		241/15	<0.12	0.06	2	>16	4	4	0.12	<0.25	<0.12	2	2	2	<0.3
		MIC50/90	<0.12/<0.12	<0.06/0.06	1/2	16/>16	4/4	2/4	0.12/0.12	<0.25/<0.25	<0.12/<0.12	<1/2	0.5/2	2/4	<0.3/<0.3
		MIC range	<0.12	<0.06–0.06	1–2	16–>16	4	2–4	0.12	<0.25	<0.12	<1–2	0.5–2	2–4	<0.3
*M. innocens*	V (4)	1010001454	0.5	<0.06	2	4	4	2	0.12	<0.25	<0.12	2	1	4	<0.3
		1010001493	<0.12	0.12	<0.25	4	4	2	0.12	<0.25	0.25	2	2	4	<0.3
		49_11	0.5	<0.06	1	>16	<0.5	<1	<0.12	<0.25	0.5	4	2	8	<0.3
		MK13	<0.12	0.12	<0.25	1	4	2	2	<0.25	0.25	<1	2	1	<0.3
		MIC50/90	<0.12/0.5	<0.06/0.12	<0.25/2	4/>16	4/4	2/2	0.12/2	<0.25/<0.25	0.25/0.5	2/4	2/2	4/8	<0.3/<0.3
		MIC range	<0.12–0.5	<0.06–0.12	<0.25–2	1–>16	<0.5–4	<1–2	<0.12–2	<0.25	<0.12–0.5	<1–4	1–2	1–8	<0.3
*M. attenuatum*	VI (2)	NLA001001166	0.25	0.12	2	16	8	1	<0.12	<0.25	<0.12	2	1	8	<0.3
		MK41	0.5	0.5	0.5	4	16	8	<0.12	<0.25	0.5	<1	4	>16	<0.3
		MIC50/90	0.25/0.5	0.12/0.5	0.5/2	4/16	8/16	1/8	<0.12/<0.12	<0.25/<0.25	<0.12/0.5	<1/2	1/4	8/>16	<0.3/<0.3
		MIC range	0.25–0.5	0.12–0.5	0.5–2	4–16	8–16	1–8	<0.12	<0.25	<0.12–0.5	<1–2	1–4	8–>16	<0.3
TOTAL	MIC50/90	0.25/1	0.12/0.5	1/2	>16/>16	4/16	2/8	<0.12/0.12	<0.25/<0.25	<0.12–0.25	2/4	1/2	4/16	<0.3/<0.3	
	MIC range	<0.12–1	<0.06–>64	<0.25–2	1–>16	<0.5–16	<1–8	<0.12–2	<0.25	<0.12–0.5	<1–4	0.25–4	1–>16	<0.3–0.6	

*Please note, that for some isolates those data were reported elsewhere (Bakuła et al., 2018c)*.

a *According to the WGS-based (MiSI method) grouping. Vertically are given the newly proposed names for each of the M. kansasii subtypes*.

b *RIF, rifampicin; CLR, clarithromycin; INH, isoniazid; EMB, ethambutol; STR, streptomycin; AMK, amikacin; SXT, co-trimoxazole; RFB, rifabutin; MXF, moxifloxacin; LZD, linezolid; CIP, ciprofloxacin; DOX, doxycycline; ETO, ethionamide*.

In conclusion, the present paper updates and extends the findings of earlier investigation on the taxonomy of *M. kansasii*. Not only does it further substantiate the delineation of new species from the *M. kansasii* group to replace the former subtypes I–VI, but consolidates the position of five of the so erected species, and provides a description of the sixth one, *M. ostraviense*, a successor of the subtype IV. By showing a close genetic relatedness, a monophyletic origin, and overlapping phenotypes, our findings support the recognition of the *M. kansasii* complex (MKC), accommodating all *M. kansasii*-derived species and *M. gastri*. Neither of the most commonly used taxonomic markers can accurately distinguish all the MKC species. Likewise, no species-specific phenotypic characteristics exist that would allow identification of the species, except the non-photochromogenicity of *M. gastri*. In the context of the previously proposed polyphasic strategy in resolving species boundaries and their interrelatedness, FAME (fatty acid methyl ester) analysis, as an adjunct typing method, might be useful (Saini et al., 2009). However, preparatory techniques for FAME analysis are typically time-consuming, laborious, and material-intensive. Furthermore, chromatography-dedicated facilities require investment in instrumentation and training, and despite their services being offered by universities and other centres, they are often less accessible than sequencing facilities, even in the developing countries.

To distinguish, most reliably, between the MKC species, and between *M. kansasii* and *M. persicum* in particular, whole-genome-based approaches should be applied.

Since no clear differences in the repertoire of the virulence-associated RD1 genes have been observed among the *M. kansasii*-derived species, the pathogenic capacity of each of these species can only be speculated based on their prevalence among the clinically relevant population. Large-scale molecular epidemiological studies are needed to gain a better understanding of the clinical significance and pathobiology of the MKC species.

### Description of *Mycobacterium ostraviense* sp. nov. Jagielski and Ulmann

*Mycobacterium ostraviense* [os.tra.vi.en'se. N.L. neut. adj. *ostraviense* pertaining to Ostravia, the Latin name of Ostrava, a city in the north-east of the Czech Republic where one of the strain was isolated].

The species name refers to the former *M. kansasii* subtype IV. Mature colonies, of rough surface and photochromogenic, are observed on Löwenstein-Jensen medium after more than 7 days of incubation at 37°C (Supplementary Figure 1). No growth occurs at 45°C and on the media containing TCH or 5% (w/v) NaCl. Similar to other MKC species, it tests positive for the semi-quantitative catalase (>45 mm) and 14-day arylsulfatase activity, while negative for niacin accumulation, tellurite reduction, and urease and pyrazynamidase activities. Unlike to other *M. kansasii*-derived species but similar to *M. gastri*, it does not reduce nitrates, nor it produces a heat-stable (68°C) catalase. Strongly resistant to ethambutol (> 16 mg/L) but susceptible to amikacin, clarithromycin, co-trimoxazole, linezolid, fluoroquinolones, and rifamycins.

The species has the same 16S rRNA sequences as *M. kansasii* (former subtype I) and *M. gastri*, yet it displays unique sequences at the *hsp65, tuf*, and *rpoB* genes, and the ITS locus. At the genomic level, it is most closely related to *M. gastri*, with pairwise ANI and GGD values of 95.2 and 0.056, respectively.

The type strain, 241/15^T^ was isolated from a sputum of a patient with no NTM disease, based in Karviná, near Ostrava, in the Moravian-Silesian Region of the Czech Republic. The type strain has been deposited in the Leibniz Institute German Collection of Microorganisms and Cell Cultures (DSMZ; Braunschweig, Germany) under the accession number DSM 110538.

## Data Availability Statement

All datasets generated for this study are included in the article/Supplementary Material.

## Ethics Statement

Analyses were based on data that did not contain any sensitive personal information. Therefore, informed consent and ethical approval were not required in like with local legislation.

## Author Contributions

TJ conceptualized and supervised the study, provided the funding, organized and integrated the data, and wrote the manuscript. PB, JL, and DS performed bioinformatic analyses including AF-ANI, GGD, and phylogenetic tree analysis. ZB performed culturing, subtyping, and phenotypic profiling of *M. kansasii* strains. BM carried out analysis on regions of difference 1-14 (RD1-14) with a homemade script Diffind. AB carried out DNA isolations for whole-genome sequencing. JD analyzed the results on the distribution of the RD1-14 genes in *M. kansasii* genomes. MD constructed the core-genome phylogenies. LP performed drug susceptibility testing. JI provided 13 *M. kansasii* strains of subtypes I-VI and critically reviewed the manuscript. MZ-D co-performed phenotypic assays.

### Conflict of Interest

The authors declare that the research was conducted in the absence of any commercial or financial relationships that could be construed as a potential conflict of interest.

## Supplementary Material

Supplementary Tables 1–6 are published inside Supplementary Data Sheet 1.

Supplementary Table 1 Pairwise similarities (%) between 16S rRNA sequences.

Supplementary Table 2 Pairwise similarities (%) between ITS sequences.

Supplementary Table 3 Pairwise similarities (%) between *hsp65* sequences.

Supplementary Table 4 Pairwise similarities (%) between *tuf* sequences.

Supplementary Table 5 Pairwise similarities (%) between *rpoB* sequences.

Supplementary Table 6 Strains (genomes) used in different genetic analyses.

Supplementary Figure 1 Macro- and micromorphology of colonies of four recently established species within the *M. kansasii* complex. Colonies photographed after 2 weeks of growth at 37°C on Löwenstein-Jensen medium, and additional 72 h post-light exposure (upper row of images). Cell morphological patterns upon Ziehl-Neelsen stain (magnification, x100), and scanning electron microscopy (magnification, x6,000). Scale bar 2 μM is applicable for *M. pseudokansasii, M. ostraviense*, and *M. innocens*, while scale bar 3 μM applies to *M. attenuatum*.

Supplementary Data Sheet 2 Distribution of region of difference 2-14 (RD2-14) genes among *M. kansasii* strains and other mycobacterial outgroup species.
