# Supplementary material for: Genomic Insights Into the Mycobacterium kansasii Complex: An Update
Source: Front Microbiol. 2020 Jan 15;10:2918. doi: 10.3389/fmicb.2019.02918 (PMC6974680; doi:10.3389/fmicb.2019.02918)
Supplement: Supplementary Table 1 — Pairwise similarities (%) between 16S rRNA sequences. [file Data_Sheet_1.docx]

**Supplementary Table 1.** Pairwise similarities (%) between 16S rRNA sequences.

| **Species/subtype^a^** | *M. kansasii* I | *M. kansasii* II+ | *M. kansasii* II | *M. persicum* | *M. kansasii* III | *M. kansasii* IV | *M. kansasii* V | *M. kansasii* VI | *M. tuberculosis* | *M. conspicuum* | *M. gastri* | *M. marinum* | *M. riyadhense* | *M. szulgai* |
| --- | --- | --- | --- | --- | --- | --- | --- | --- | --- | --- | --- | --- | --- | --- |
| *M. kansasii* I | **99.2-100.0** |  |  |  |  |  |  |  |  |  |  |  |  |  |
| *M. kansasii* II+ | 99.2-100.0 | **100.0** |  |  |  |  |  |  |  |  |  |  |  |  |
| *M. kansasii* II | 99.2-100.0 | 100.0 | **100.0** |  |  |  |  |  |  |  |  |  |  |  |
| *M. persicum* | 99.2-100.0 | 100.0 | 100.0 | **100.0** |  |  |  |  |  |  |  |  |  |  |
| *M. kansasii* III | 99.2-99.9 | 99.9 | 99.9 | 99.9 | **100.0** |  |  |  |  |  |  |  |  |  |
| *M. kansasii* IV | 99.3-100.0 | 99.3 | 99.3 | 99.3 | 99.3 | **100.0** |  |  |  |  |  |  |  |  |
| *M. kansasii* V | 99.2-99.8 | 99.2 | 99.2 | 99.2 | 99.2 | 99.8 | **100.0** |  |  |  |  |  |  |  |
| *M. kansasii* VI | 99.0-99.8 | 99.8 | 99.8 | 99.8 | 99.8 | 99.2 | 99.0 | **99.8-100.0** |  |  |  |  |  |  |
| *M. tuberculosis* | 98.1-98.6 | 98.1 | 98.1 | 98.1 | 98.1 | 98.6 | 98.5 | 98.0 | –* |  |  |  |  |  |
| *M. conspicuum* | 98.2-98.4 | 98.4 | 98.4 | 98.4 | 98.5 | 98.3 | 98.2 | 98.5 | 97.8 | –* |  |  |  |  |
| *M. gastri* | 99.3-100.0 | 99.3 | 99.3 | 99.3 | 99.3 | 100.0 | 99.8 | 99.2 | 98.6 | 98.3 | –* |  |  |  |
| *M. marinum* | 98.1-98.6 | 98.1 | 98.1 | 98.1 | 98.2 | 98.6 | 98.5 | 98.2-98.3 | 99.1 | 98.1 | 98.6 | –* |  |  |
| *M. riyadhense* | 98.7-98.9 | 98.9 | 98.9 | 98.9 | 99.0 | 98.8 | 98.7 | 99.0-99.1 | 98.3 | 98.6 | 98.8 | 98.5 | –* |  |
| *M. szulgai* | 98.8-99.0 | 99.0 | 99.0 | 99.0 | 99.1 | 99.0 | 98.8 | 99.1 | 98.4 | 98.8 | 99.0 | 98.5 | 99.6 | –* |

^a^ Each *M. kansasii* subtype was represented by a group of 2–31 strains (genomes). The *M. kansasii* II group included 7 strains (2193, NLA001001128, B11073207, B11063838, 1010001469, H47, and H48); the *M. persicum* group included 4 strains [AFPC-000227 (T) (Shahraki et al., 2017), MK4, MK15, and MK42 (Tagini et al., 2019)]; the *M. kansasii* II+ group incorporated strains of both *M. kansasii* II and *M. persicum* groups (11 strains in total). The *M. kansasii* groups III, V, and VI included strains of respective genotypes collected for this study as well as strains which had elsewhere been designated as *M. pseudokansasii* (MK21, MK35, and MK142), *M. innocens* (MK13), and *M. attenuatum* (MK41, MK136, MK191), accordingly (Tagini et al., 2019). In addition, genomes of single *M. tuberculosis*, *M. conspicuum*, *M. gastri*, *M. marinum*, *M. riyadhense*, and *M. szulgai* strains were analysed. See **Suppl. Table 6**.

^*^, The values are not given, since only one genome sequence per species was analyzed. Similarities calculated within the groups of strains (genomes) are indicated in bold.

**Supplementary Table 2.** Pairwise similarities (%) between ITS sequences.

| **Species/subtype^a^** | *M. kansasii* I | *M. kansasii* II+ | *M. kansasii* II | *M. persicum* | *M. kansasii* III | *M. kansasii* IV | *M. kansasii* V | *M. kansasii* VI | *M. tuberculosis* | *M. conspicuum* | *M. gastri* | *M. marinum* | *M. riyadhense* | *M. szulgai* |
| --- | --- | --- | --- | --- | --- | --- | --- | --- | --- | --- | --- | --- | --- | --- |
| *M. kansasii* I | **92.1-100.0** |  |  |  |  |  |  |  |  |  |  |  |  |  |
| *M. kansasii* II+ | 91.7-100.0 | **99.2-100.0** |  |  |  |  |  |  |  |  |  |  |  |  |
| *M. kansasii* II | 91.7-100.0 | 99.2-100.0 | **99.2-100.0** |  |  |  |  |  |  |  |  |  |  |  |
| *M. persicum* | 92.1-100.0 | 99.2-100.0 | 99.2-100.0 | **99.6-100.0** |  |  |  |  |  |  |  |  |  |  |
| *M. kansasii* III | 84.7-88.2 | 87.5-88.6 | 87.5-88.6 | 87.9-88.6 | **96.4-100.0** |  |  |  |  |  |  |  |  |  |
| *M. kansasii* IV | 83.6-85.6 | 85.2-85.9 | 85.2-85.9 | 85.6-85.9 | 81.2-82.2 | **100.0** |  |  |  |  |  |  |  |  |
| *M. kansasii* V | 84.1-86.5 | 86.1-86.8 | 86.1-86.8 | 86.5-86.8 | 81.8-82.8 | 90.2 | **99.3-100.0** |  |  |  |  |  |  |  |
| *M. kansasii* VI | 85.1-86.8 | 86.5-87.2 | 86.5-87.2 | 86.8-87.2 | 93.5-95.3 | 81.2 | 81.8 | **100.0** |  |  |  |  |  |  |
| *M. tuberculosis* | 83.3-84.4 | 84.4-84.8 | 84.4-84.8 | 84.4-84.8 | 80.5-81.9 | 79.9 | 79.1 | 81.9 | –* |  |  |  |  |  |
| *M. conspicuum* | 78.4-81.6 | 81.2-81.6 | 81.2-81.6 | 81.2-81.6 | 79.1 | 79.7 | 81.2 | 77.8 | 77.2 | –* |  |  |  |  |
| *M. gastri* | 83.9-85.6 | 85.2-85.9 | 85.2-85.9 | 85.6-85.9 | 80.5-82.2 | 97.9 | 90.2 | 80.5 | 80.2 | 80.4 | –* |  |  |  |
| *M. marinum* | 88.1-88.8 | 87.8-88.5 | 87.8-88.5 | 87.8-88.1 | 85.7-86.1 | 82.8 | 83.1 | 85.4 | 82.6 | 81.1 | 82.8 | –* |  |  |
| *M. riyadhense* | 90.7-91.8 | 91.4-92.1 | 91.4-92.1 | 91.8-92.1 | 83.8-84.5 | 85.7 | 85.2 | 84.5 | 88.0 | 81.0 | 85.3 | 88.6 | –* |  |
| *M. szulgai* | 90.3-94.9 | 94.6-95.3 | 94.6-95.3 | 94.9-95.3 | 85.8-86.1 | 83.9 | 83.1 | 86.1 | 84.4 | 79.2 | 84.8 | 87.4 | 90.7 | –* |

^a^ Each *M. kansasii* subtype was represented by a group of 2–31 strains (genomes). The *M. kansasii* II group included 7 strains (2193, NLA001001128, B11073207, B11063838, 1010001469, H47, and H48); the *M. persicum* group included 4 strains [AFPC-000227 (T) (Shahraki et al., 2017), MK4, MK15, and MK42 (Tagini et al., 2019)]; the *M. kansasii* II+ group incorporated strains of both *M. kansasii* II and *M. persicum* groups (11 strains in total). The *M. kansasii* groups III, V, and VI included strains of respective genotypes collected for this study as well as strains which had elsewhere been designated as *M. pseudokansasii* (MK21, MK35, and MK142), *M. innocens* (MK13), and *M. attenuatum* (MK41, MK136, MK191), accordingly (Tagini et al., 2019). In addition, genomes of single *M. tuberculosis*, *M. conspicuum*, *M. gastri*, *M. marinum*, *M. riyadhense*, and *M. szulgai* strains were analysed. See **Suppl. Table 6**.

^*^, The values are not given, since only one genome sequence per species was analyzed. Similarities calculated within the groups of strains (genomes) are indicated in bold.

**Supplementary Table 3.** Pairwise similarities (%) between *hsp65* sequences.

| **Species/subtype^a^** | *M. kansasii* I | *M. kansasii* II+ | *M. kansasii* II | *M. persicum* | *M. kansasii* III | *M. kansasii* IV | *M. kansasii* V | *M. kansasii* VI | *M. tuberculosis* | *M. conspicuum* | *M. gastri* | *M. marinum* | *M. riyadhense* | *M. szulgai* |
| --- | --- | --- | --- | --- | --- | --- | --- | --- | --- | --- | --- | --- | --- | --- |
| *M. kansasii* I | **97.3-100.0** |  |  |  |  |  |  |  |  |  |  |  |  |  |
| *M. kansasii* II+ | 96.7-99.8 | **99.5-100.0** |  |  |  |  |  |  |  |  |  |  |  |  |
| *M. kansasii* II | 96.7-99.8 | 99.5-100.0 | **99.5-100.0** |  |  |  |  |  |  |  |  |  |  |  |
| *M. persicum* | 96.8-99.5 | 99.5-100.0 | 99.5-100.0 | **100.0** |  |  |  |  |  |  |  |  |  |  |
| *M. kansasii* III | 95.3-96.7 | 96.1-96.5 | 96.1-96.5 | 96.1-96.2 | **99.8-100.0** |  |  |  |  |  |  |  |  |  |
| *M. kansasii* IV | 97.2-97.8 | 97.2-97.6 | 97.2-97.6 | 97.3-97.3 | 95.9-96.1 | **100.0** |  |  |  |  |  |  |  |  |
| *M. kansasii* V | 94.8-95.4 | 94.2-94.8 | 94.2-94.8 | 94.4-94.5 | 93.4-93.7 | 94.5-94.7 | **99.5-100.0** |  |  |  |  |  |  |  |
| *M. kansasii* VI | 95.9-96.5 | 95.6-96.4 | 95.6-96.4 | 95.8-96.1 | 95.6-96.1 | 95.9-96.2 | 94.0-94.5 | **99.6-100.0** |  |  |  |  |  |  |
| *M. tuberculosis* | 91.3-92.3 | 92.0-92.2 | 92.0-92.2 | 92.2 | 90.3-90.5 | 91.9 | 92.2 | 91.3-91.6 | –* |  |  |  |  |  |
| *M. conspicuum* | 94.5-94.7 | 94.4-94.5 | 94.4-94.5 | 94.4 | 94.7-94.8 | 94.2 | 93.4-93.6 | 93.4-93.7 | 90.9 | –* |  |  |  |  |
| *M. gastri* | 97.5-97.9 | 97.3-97.8 | 97.3-97.8 | 97.5 | 95.8-95.9 | 97.8 | 94.0-94.2 | 95.9-96.2 | 91.4 | 94.0 | –* |  |  |  |
| *M. marinum* | 93.7-94.2 | 93.9-94.4 | 93.9-94.4 | 94.0 | 92.8-93.0 | 93.6 | 93.1-93.4 | 93.4-93.7 | 91.6 | 93.9 | 94.0 | –* |  |  |
| *M. riyadhense* | 93.0-93.1 | 92.8-93.1 | 92.8-93.1 | 92.8 | 90.9-91.1 | 92.7 | 93.4-93.7 | 92.0-92.3 | 90.6 | 93.3 | 92.5 | 93.4 | –* |  |
| *M. szulgai* | 92.8-93.4 | 92.7-92.8 | 92.7-92.8 | 92.7 | 93.1 | 93.0 | 93.0-93.3 | 92.3-92.7 | 90.5 | 96.2 | 92.8 | 94.0 | 93.6 | –* |

^a^ Each *M. kansasii* subtype was represented by a group of 2–31 strains (genomes). The *M. kansasii* II group included 7 strains (2193, NLA001001128, B11073207, B11063838, 1010001469, H47, and H48); the *M. persicum* group included 4 strains [AFPC-000227 (T) (Shahraki et al., 2017), MK4, MK15, and MK42 (Tagini et al., 2019)]; the *M. kansasii* II+ group incorporated strains of both *M. kansasii* II and *M. persicum* groups (11 strains in total). The *M. kansasii* groups III, V, and VI included strains of respective genotypes collected for this study as well as strains, which had elsewhere been designated as *M. pseudokansasii* (MK21, MK35, and MK142), *M. innocens* (MK13), and *M. attenuatum* (MK41, MK136, MK191), accordingly (Tagini et al., 2019). In addition, genomes of single *M. tuberculosis*, *M. conspicuum*, *M. gastri*, *M. marinum*, *M. riyadhense*, and *M. szulgai* strains were analysed. See **Suppl. Table 6**.

^*^, The values are not given, since only one genome sequence per species was analyzed. Similarities calculated within the groups of strains (genomes) are indicated in bold.

**Supplementary Table 4.** Pairwise similarities (%) between *tuf* sequences.

| **Species/subtype^a^** | *M. kansasii* I | *M. kansasii* II+ | *M. kansasii* II | *M. persicum* | *M. kansasii* III | *M. kansasii* IV | *M. kansasii* V | *M. kansasii* VI | *M. tuberculosis* | *M. conspicuum* | *M. gastri* | *M. marinum* | *M. riyadhense* | *M. szulgai* | |
| --- | --- | --- | --- | --- | --- | --- | --- | --- | --- | --- | --- | --- | --- | --- | --- |
| *M. kansasii* I | **97.3-100.0** |  |  |  |  |  |  |  |  |  |  |  |  |  | |
| *M. kansasii* II+ | 97.3-100.0 | **100.0** |  |  |  |  |  |  |  |  |  |  |  |  | |
| *M. kansasii* II | 97.3-100.0 | 100.0 | **100.0** |  |  |  |  |  |  |  |  |  |  |  | |
| *M. persicum* | 97.3-100.0 | 100.0 | 100.0 | **100.0** |  |  |  |  |  |  |  |  |  |  | |
| *M. kansasii* III | 97.0-97.4 | 97.4 | 97.4 | 97.4 | **100.0** |  |  |  |  |  |  |  |  |  | |
| *M. kansasii* IV | 97.6-98.2 | 98.2 | 98.2 | 98.2 | 97.7 | **100.0** |  |  |  |  |  |  |  |  | |
| *M. kansasii* V | 97.5-98.1 | 97.9-98.1 | 97.9-98.1 | 97.9-98.1 | 97.4-97.6 | 98.2-98.3 | **99.8-100.0** |  |  |  |  |  |  |  | |
| *M. kansasii* VI | 94.9-95.2 | 94.9-95.0 | 94.9-95.0 | 94.9-95.0 | 94.5-94.6 | 95.7-95.8 | 95.4-95.5 | **99.9-100.0** |  |  |  |  |  |  | |
| *M. tuberculosis* | 92.2-92.8 | 92.8 | 92.8 | 92.8 | 92.5 | 93.4 | 92.7-92.9 | 92.3-92.4 | –* |  |  |  |  |  | |
| *M. conspicuum* | 93.3-93.7 | 93.7 | 93.7 | 93.7 | 92.9 | 93.7 | 93.3-93.5 | 92.9-93.0 | 92.2 | –* |  |  |  |  | |
| *M. gastri* | 96.6-97.5 | 97.5 | 97.5 | 97.5 | 97.2 | 97.8 | 97.1-97.2 | 94.5-94.6 | 92.5 | 93.1 | –* |  |  |  | |
| *M. marinum* | 92.2-92.4 | 92.3 | 92.3 | 92.3 | 91.8 | 92.7 | 92.1-92.2 | 91.6-91.7 | 90.5 | 93.3 | 91.9 | –* |  |  | |
| *M. riyadhense* | 94.1-94.3 | 94.3 | 94.3 | 94.3 | 93.9 | 94.4 | 93.5-93.7 | 93.2-93.3 | 93.8 | 94.4 | 93.8 | 92.1 | –* |  | |
| *M. szulgai* | 93.8-94.0 | 93.8 | 93.8 | 93.8 | 93.8 | 93.8 | 93.6-93.8 | 92.9-93.0 | 92.9 | 93.8 | 93.4 | 92. | 94.4 | –* | |
|  | | | | | | | | | | | | | | |  |

^a^ Each *M. kansasii* subtype was represented by a group of 2–31 strains (genomes). The *M. kansasii* II group included 7 strains (2193, NLA001001128, B11073207, B11063838, 1010001469, H47, and H48); the *M. persicum* group included 4 strains [AFPC-000227 (T) (Shahraki et al., 2017), MK4, MK15, and MK42 (Tagini et al., 2019)]; the *M. kansasii* II+ group incorporated strains of both *M. kansasii* II and *M. persicum* groups (11 strains in total). The *M. kansasii* groups III, V, and VI included strains of respective genotypes collected for this study as well as strains, which had elsewhere been designated as *M. pseudokansasii* (MK21, MK35, and MK142), *M. innocens* (MK13), and *M. attenuatum* (MK41, MK136, MK191), accordingly (Tagini et al., 2019). In addition, genomes of single *M. tuberculosis*, *M. conspicuum*, *M. gastri*, *M. marinum*, *M. riyadhense*, and *M. szulgai* strains were analysed. See **Suppl. Table 6**.

^*^, The values are not given, since only one genome sequence per species was analyzed. Similarities calculated within the groups of strains (genomes) are indicated in bold.

**Supplementary Table 5.** Pairwise similarities (%) between *rpoB* sequences.

| **Species/subtype^a^** | *M. kansasii* I | *M. kansasii* II+ | *M. kansasii* II | *M. persicum* | *M. kansasii* III | *M. kansasii* IV | *M. kansasii* V | *M. kansasii* VI | *M. tuberculosis* | *M. conspicuum* | *M. gastri* | *M. marinum* | *M. riyadhense* | *M. szulgai* |
| --- | --- | --- | --- | --- | --- | --- | --- | --- | --- | --- | --- | --- | --- | --- |
| *M. kansasii* I | **95.3-100.0** |  |  |  |  |  |  |  |  |  |  |  |  |  |
| *M. kansasii* II+ | 95.2-100.0 | **99.8-100.0** |  |  |  |  |  |  |  |  |  |  |  |  |
| *M. kansasii* II | 95.2-100.0 | 99.8-100.0 | **99.8-100.0** |  |  |  |  |  |  |  |  |  |  |  |
| *M. persicum* | 95.2-99.9 | 99.8-100.0 | 99.8-100.0 | **99.8-99.9** |  |  |  |  |  |  |  |  |  |  |
| *M. kansasii* III | 94.7-95.5 | 94.7 | 94.7 | 94.7 | **99.9-100.0** |  |  |  |  |  |  |  |  |  |
| *M. kansasii* IV | 93.4-94.1 | 93.4 | 93.4 | 93.4 | 93.4 | **100.0** |  |  |  |  |  |  |  |  |
| *M. kansasii* V | 95.3-97.9 | 95.3 | 95.3 | 95.3 | 95.6-95.7 | 94.3 | **99.5-100.0** |  |  |  |  |  |  |  |
| *M. kansasii* VI | 92.6-93.1 | 93.0-93.1 | 93.0-93.1 | 93.0-93.1 | 94.0-94.2 | 91.9-92.0 | 92.6-92.7 | **99.9-100.0** |  |  |  |  |  |  |
| *M. tuberculosis* | 87.1-88.6 | 87.1 | 87.1 | 87.1 | 87.9 | 87.2 | 88.4-88.5 | 88.2-88.3 | –* |  |  |  |  |  |
| *M. conspicuum* | 89.7-90.4 | 89.7 | 89.7 | 89.7 | 90.3 | 89.0 | 90.5-90.6 | 90.3 | 89.4 | –* |  |  |  |  |
| *M. gastri* | 94.3-94.6 | 94.5-94.6 | 94.6 | 94.5-94.6 | 93.8 | 95.9 | 94.5-94.6 | 93.5-93.6 | 87.6 | 90.1 | –* |  |  |  |
| *M. marinum* | 88.4-89.3 | 88.4-88.5 | 88.4 | 88.4-88.5 | 89.0 | 88.0 | 89.3-89.4 | 89.3 | 89.3 | 90.5 | 88.9 | –* |  |  |
| *M. riyadhense* | 88.4-89.5 | 88.4 | 88.4 | 88.4 | 89.3 | 88.2 | 89.5 | 89.3 | 90.0 | 91.6 | 89.2 | 90.6 | –* |  |
| *M. szulgai* | 89.0-89.4 | 89.0 | 89.0 | 89.0 | 89.3 | 88.2 | 89.6 | 89.7-89.8 | 88.8 | 91.7 | 89.5 | 90.5 | 91.5 | –* |
|  |  |  |  |  |  |  |  |  |  |  |  |  |  |  |

^a^ Each *M. kansasii* subtype was represented by a group of 2–31 strains (genomes). The *M. kansasii* II group included 7 strains (2193, NLA001001128, B11073207, B11063838, 1010001469, H47, and H48); the *M. persicum* group included 4 strains [AFPC-000227 (T) (Shahraki et al., 2017), MK4, MK15, and MK42 (Tagini et al., 2019)]; the *M. kansasii* II+ group incorporated strains of both *M. kansasii* II and *M. persicum* groups (11 strains in total). The *M. kansasii* groups III, V, and VI included strains of respective genotypes collected for this study as well as strains, which had elsewhere been designated as *M. pseudokansasii* (MK21, MK35, and MK142), *M. innocens* (MK13), and *M. attenuatum* (MK41, MK136, MK191), accordingly (Tagini et al., 2019). In addition, genomes of single *M. tuberculosis*, *M. conspicuum*, *M. gastri*, *M. marinum*, *M. riyadhense*, and *M. szulgai* strains were analysed. See **Suppl. Table 6**.

^*^, The values are not given, since only one genome sequence per species was analyzed. Similarities calculated within the groups of strains (genomes) are indicated in bold.

**Supplementary Table 6.** Strains (genomes) used in different genetic analyses.

| **No.** | **Species/genotype^a^** | **Strain^b^** | **GenBank no.** | **Type of analysis^c^** | | | | |
| --- | --- | --- | --- | --- | --- | --- | --- | --- |
|  |  |  |  | **AF/ANI/MiSI** | **GGD** | **CGP** | **RD1** | **PS** |
|  | *M. kansasii* I | NLA001000521 | MWKY01 | + | + | + | + | + |
|  | *M. kansasii* I | ATCC25221 | CP019885 | + | + | + | + | + |
|  | *M. kansasii* I | NLA001000927 | CP019883 | + | + | + | + | + |
|  | *M. kansasii* I | NLA001000449 | CP019884 | + | + | + | + | + |
|  | *M. kansasii* I | 6200 | CP019886 | + | + | + | + | + |
|  | *M. kansasii* I | 7728 | CP019888 | + | + | + | + | + |
|  | *M. kansasii* I | 7744 | CP019887 | + | + | + | + | + |
|  | *M. kansasii* I | 1010001495^†^ | LWCL01 | + | + | + | + | + |
|  | *M. kansasii* I | K4 | NKQW01 | + | + | + | + | + |
|  | *M. kansasii* I | K14^*^ | NKQY01 | + | + | + | + | + |
|  | *M. kansasii* I | K19^*^ | NKQX01 | + | + | + | + | + |
|  | *M. kansasii* I | ATCC12478^†^ | CP0006835 | + | + | + | + | + |
|  | *M. kansasii* I | 824 | CP009483 | + | + | – | + | + |
|  | *M. kansasii* I | BR3657 | PQOL01 | + | + | – | + | + |
|  | *M. kansasii* I | BR6849 | PQOP01 | + | + | – | + | + |
|  | *M. kansasii* I | SMC1 | JNDJ01 | + | + | – | + | + |
|  | *M. kansasii* I | BR8837 | PQOV01 | + | + | – | + | + |
|  | *M. kansasii* I | BR6498 | PQOM01 | + | + | – | + | + |
|  | *M. kansasii* I | 11_3813 | MVBM01 | + | + | – | + | + |
|  | *M. kansasii* I | BR6884 | PQOO01 | + | + | – | + | + |
|  | *M. kansasii* I | BR10742 | PQOR01 | + | + | – | + | + |
|  | *M. kansasii* I | BR1580 | PQOS01 | + | + | – | + | + |
|  | *M. kansasii* I | BR4404 | PQOT01 | + | + | – | + | + |
|  | *M. kansasii* I | BR8839 | PQOW01 | + | + | – | + | + |
|  | *M. kansasii* I | 662 | CP009481 | + | + | – | + | + |
|  | *M. kansasii* I | BR7287 | PQON01 | + | + | – | + | + |
|  | *M. kansasii* I | BR10953 | PQOQ01 | + | + | – | + | + |
|  | *M. kansasii* I | BR8835 | PQOU01 | + | + | – | + | + |
|  | *M. kansasii* I | 11_3469 | MVBN01 | + | + | – | + | + |
|  | *M. kansasii* I | MK40^†^ | UPHI01 | + | + | + | + | + |
|  | *M. kansasii* I | MK7^†^ | UPHJ01 | + | + | + | + | + |
|  | *M. kansasii* II | 2193 | MWQA01 | + | + | + | + | + |
|  | *M. kansasii* II | NLA001001128 | MWKX01 | + | + | + | + | + |
|  | *M. kansasii* II | B11073207 | MWKZ01 | + | + | + | + | + |
|  | *M. kansasii* II | B11063838 | MWKV01 | + | + | + | + | + |
|  | *M. kansasii* II | 1010001469^†^ | LWCM01 | + | + | + | + | + |
|  | *M. kansasii* II | H47 | NKRA01 | + | + | + | + | + |
|  | *M. kansasii* II | H48 | NKQZ01 | + | + | + | + | + |
|  | *M. persicum* | AFPC-000227^†^ | MVIF01 | + | + | + | + | + |
|  | *M. persicum* | MK15^†^ | UPHK01 | + | + | + | + | + |
|  | *M. persicum* | MK4^†^ | UPHM01 | + | + | + | + | + |
|  | *M. persicum* | MK42^†^ | UPHL01 | + | + | – | + | + |
|  | *M. kansasii* III | 14/15 | NKRB01 | + | + | + | + | + |
|  | *M. kansasii* III | 174/15 | NKRD01 | + | + | + | + | + |
|  | *M. kansasii* III | 1010001468^†^ | LWCJ01 | + | + | + | + | + |
|  | *M. kansasii* III | 732 | JANZ01 | + | + | – | + | + |
|  | *M. pseudokansasii* | MK142^†^ | UPHU01 | + | + | + | + | + |
|  | *M. pseudokansasii* | MK21^†^ | UPHO01 | + | + | + | + | + |
|  | *M. pseudokansasii* | MK35^†^ | UPHN01 | + | + | + | + | + |
|  | *M. kansasii* IV | 1010001458^†^ | LWCI01 | + | + | + | + | + |
|  | *M. kansasii* IV | 241/15 | NKRE01 | + | + | + | + | + |
|  | *M. kansasii* V | 1010001454^†^ | LWCH01 | + | + | + | + | + |
|  | *M. kansasii* V | 1010001493^†^ | LWCK01 | + | + | + | + | + |
|  | *M. kansasii* V | 49/11 | NKRC01 | + | + | + | + | + |
|  | *M. innocens* | MK13^†^ | UPHQ01 | + | + | + | + | + |
|  | *M. kansasii* VI | NLA001001166 | MWKW01 | + | + | + | + | + |
|  | *M. attenuatum* | MK41^†^ | UPHT01 | + | + | + | + | + |
|  | *M. attenuatum* | MK136^†^ | UPHP01 | + | + | + | + | + |
|  | *M. attenuatum* | MK191^†^ | UPHS01 | + | + | + | + | + |
|  | *M. tuberculosis* | H37Rv^†^ | NC_000962 | + | + | + | – | + |
|  | *M. tuberculosis* | Beijing-like | CP017597 | – | – | – | + | – |
|  | *M. tuberculosis* | BTB12-313 | JKCK01 | + | – | – | – | – |
|  | *M. tuberculosis* | 323 | CP010873 | + | – | – | – | – |
|  | *M. tuberculosis* | EAI5 | NC_021740 | + | – | – | – | – |
|  | *M. tuberculosis* | F11 | NC_009565 | + | – | – | – | – |
|  | *M. tuberculosis* | KIT87190 | CP007809 | + | – | – | – | – |
|  | *M. africanum* | GM041182 | NC_015758 | + | + | – | + | – |
|  | *M. africanum* | K85 | ACHQ01 | + | – | – | – | – |
|  | *M. africanum* | MAL010070 | JLAZ01 | + | – | – | – | – |
|  | *M. africanum* | MAL010084 | JKXR01 | + | – | – | – | – |
|  | *M. africanum* | UT307 | NZ_CP014617 | + | – | – | – | – |
|  | *M. bovis* | 09_1191 | JPFP01 | + | + | – | + | – |
|  | *M. bovis* | 50 | JQEU01 | + | – | – | – | – |
|  | *M. bovis* | 534 | JQEM01 | + | – | – | – | – |
|  | *M. bovis* | B_3222 | LNOF01 | + | – | – | – | – |
|  | *M. bovis* | MB1 | CDHF01 | + | – | – | – | – |
|  | *M. caprae* | Allgaeu | CP016401 | + | + | – | + | – |
|  | *M. microti* | 12 | CP010333 | + | + | – | + | – |
|  | *M. conspicuum* | DSM44136 | LQOR01.1 | + | + | + | + | + |
|  | *M. gastri* | DSM43505^†^ | LQOX01 | + | + | + | + | + |
|  | *M. marinum* | E11^†^ | HG917972 | + | + | + | + | + |
|  | *M. riyadhense* | DSM45176 | LQPQ01 | + | + | + | + | + |
|  | *M. szulgai* | DSM44166^†^ | LQPW01 | + | + | + | + | + |

^a^ According to the WGS-based (MiSI method) grouping.

^b^ *M. kansasii* strains K14 and K19 had originally been described as atypical type IIb (*), based on the PCR-RFLP/PCR-sequencing analysis of the *hsp65*, *rpoB*, and/or *tuf* genes; (†), *M. kansasii* strains whose genomes were analysed by Tagini et al (2019).

^c^ Performance of a given analysis was indicated with a plus sign (+). MiSI, Microbial Species Identifier; GGD, genome-to-genome distance; CPG, core-genome phylogeny; RD1, region of difference 1; PS, pairwise gene sequence similarities.
