# Supplementary figures and images for: Genomic Insights Into the Mycobacterium kansasii Complex: An Update
Source: Front Microbiol. 2020 Jan 15;10:2918. doi: 10.3389/fmicb.2019.02918 (PMC6974680; doi:10.3389/fmicb.2019.02918)

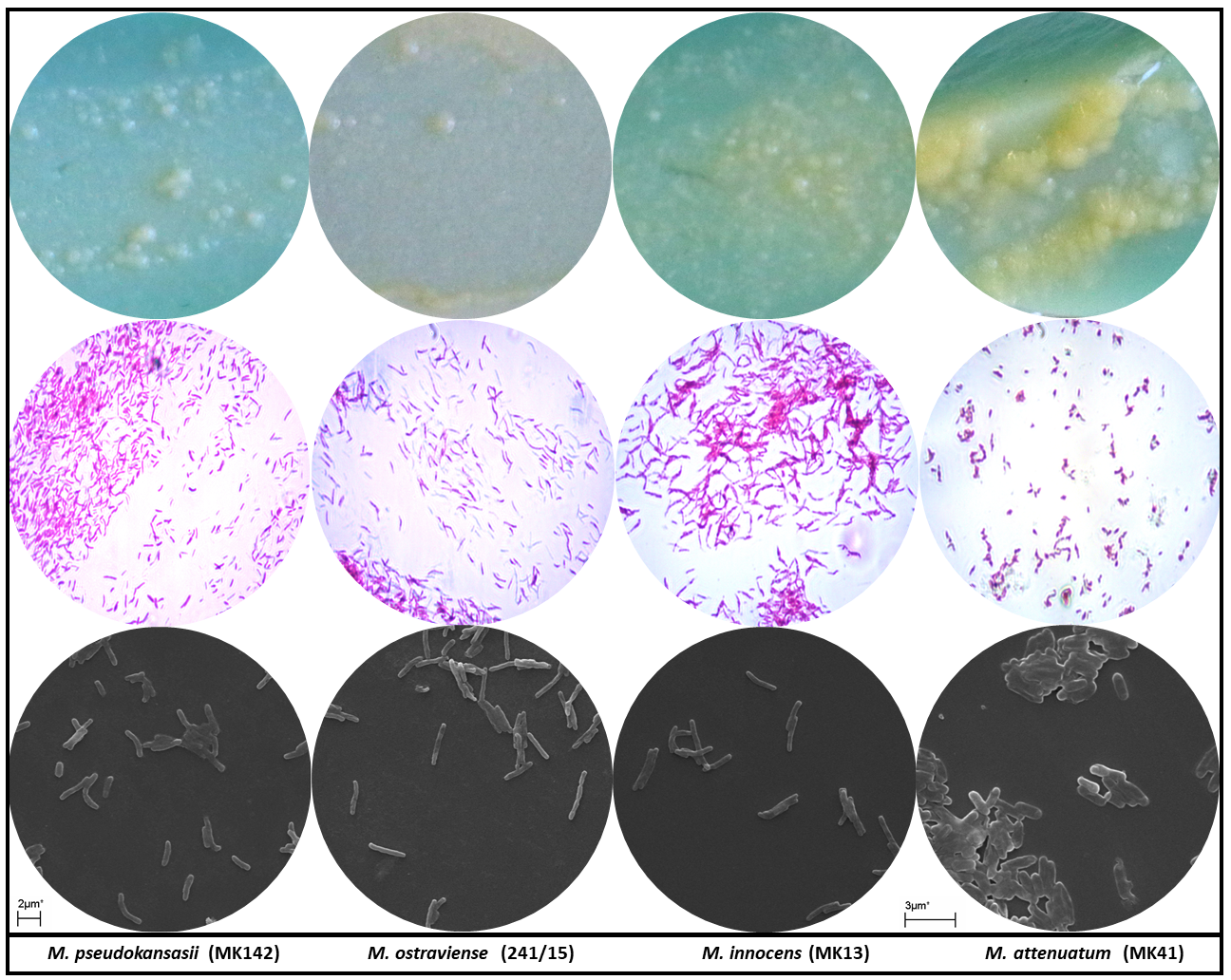

Supplement: Supplementary Figure 1 — Macro- and micromorphology of colonies of four recently established species within the M. kansasii complex. Colonies photographed after 2 weeks of growth at 37°C on Löwenstein-Jensen medium, and additional 72 h post-light exposure (upper row of images). Cell morphological patterns upon Ziehl-Neelsen stain (magnification, x100), and scanning electron microscopy (magnification, x6,000). Scale bar 2 μM is applicable for M. pseudokansasii, M. ostraviense, and M. innocens, while scale bar 3 μM applies to M. attenuatum. [file Image_1.TIF]
